# Rectification of planar orientation angle switches behavior and replenishes contractile junctions

**DOI:** 10.1083/jcb.202309069

**Published:** 2025-01-23

**Authors:** Katie Linvill, Liam J. Russell, Timothy E. Vanderleest, Hui Miao, Yi Xie, J. Todd Blankenship, Dinah Loerke

**Affiliations:** 1 Department of Physics and Astronomy, University of Denver, Denver, CO, USA; 2 Department of Biological Sciences, University of Denver, Denver, CO, USA

## Abstract

In the early *Drosophila* embryo, germband elongation is driven by oriented cell intercalation through t1 transitions, where vertical (dorsal–ventral aligned) interfaces contract and then resolve into new horizontal (anterior–posterior aligned) interfaces. Here, we show that contractile events produce a continuous “rectification” of cell interfaces, in which interfaces systematically rotate toward more vertical orientations. As interfaces rotate, their behavior transitions from elongating to contractile regimes, indicating that the planar polarized identities of cell–cell interfaces are continuously re-interpreted in time depending on their orientation angle. Rotating interfaces acquire higher levels of Myosin II motor proteins as they become more vertical, while disruptions to the contractile molecular machinery reduce the rates of rotation. Through this angle rectification, the available pool of contractile interfaces is continuously replenished, as new interfaces acquire a contractile identity through rotation. Thus, individual cells acquire additional interfaces that are capable of undergoing t1 transitions, allowing cells to participate in multiple staggered rounds of intercalation events.

## Teaser

In the early *Drosophila* embryo, germband extension requires positional switches of neighboring epithelial cells through remodeling of cell–cell junctions. Linvill et al. find that junctions systematically rotate from a horizontal towards a vertical orientation. During this process, rotating interfaces acquire myosin and newly initialized contractile identities, which allows for multiple rounds of intercalation.

## Introduction

Elongation along a primary body axis is a key morphogenetic process that occurs in many organisms during embryonic development. This elongation process often occurs through the so-called “convergent extension” of epithelial tissues, which consists of the tissue narrowing in one dimension while simultaneously extending in the perpendicular dimension. At the cellular level, this process is often driven by the coordinated and directional intercalation of neighboring cells (Irvine and Wieschaus, 1994; Keller et al., 2000; Wallingford et al., 2002; Keller, 2002; Nikolaidou and Barrett, 2005; Solnica-Krezel, 2005). One system in which this intercalation occurs is during the convergent extension of the *Drosophila* germband (Guillot and Lecuit, 2013; Loerke and Blankenship, 2020). In this system, within ∼45 min of development, the germband epithelial tissue more than doubles in length along the anterior–posterior (AP) axis—i.e., into the organism’s head-to-tail direction—while simultaneously narrowing in the dorsal–ventral (DV) direction (Irvine and Wieschaus, 1994).

**Figure 1.**
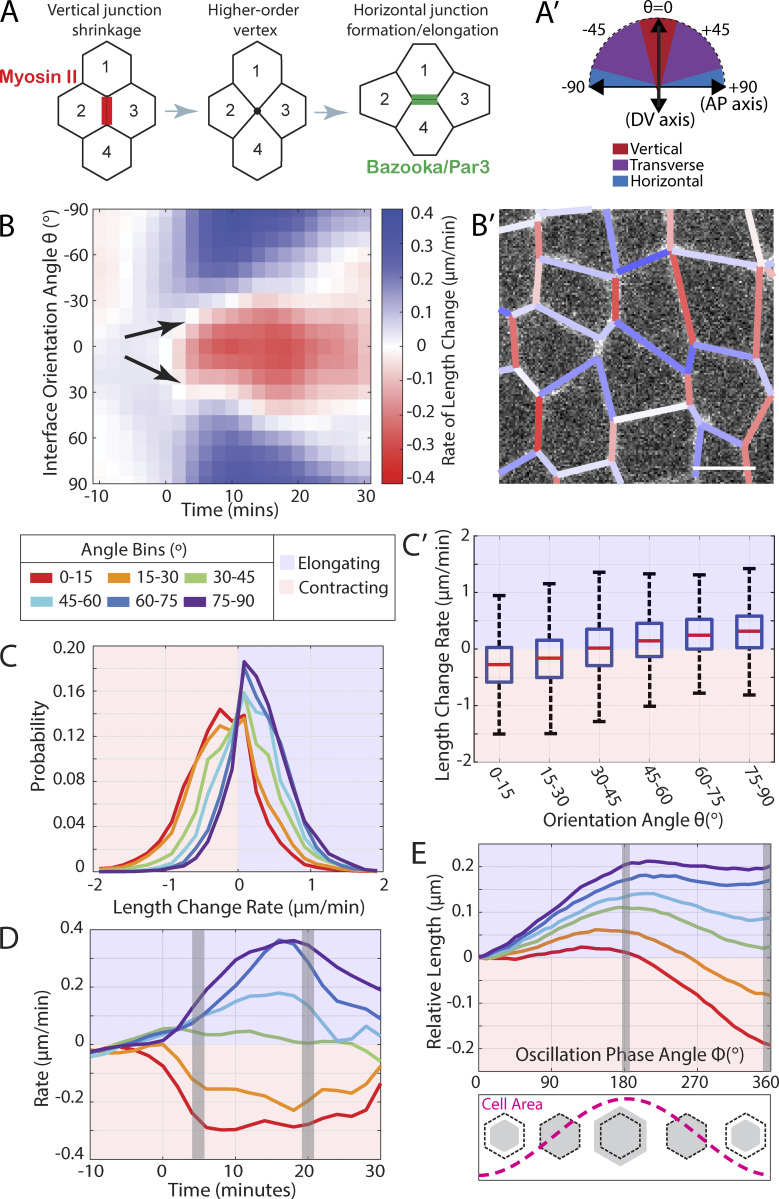
**Within a given population of interfaces, planar polarized interface behaviors occur on a continuum with interface angle. (A)** Schematic of a typical t1 transition, with Myosin II localized to vertical (DV-oriented) interfaces and Bazooka/Par3 localized to horizontal (AP-oriented) interfaces. **(A′)** Schematic of embryo coordinate system, with AP/DV axis and interface angles indicated. **(B)** Heat map of the rate of change in interface length (red: contraction; blue: elongation) as a function of time t and interface orientation angle θ. Data is averaged from *n* = 2,940 unique interfaces from k = 3 embryos; time point t = 0 corresponds to the onset of germband extension, and orientation angle θ = 0° corresponds to vertical interfaces. **(B′)** Sample image of cells, with interfaces color-coded for their measured contraction/elongation rate; scale bar 5 µm. **(C)** Distribution of measured interface contraction/elongation rates for different interface orientation angle bins. Same data set as B, constrained to the time window t = 5–20 min after initiation of germband extension. **(C′)** Same data as C is shown as a box plot. **(D)** Mean interface contraction/elongation rates as a function of developmental time t, for different interface orientation angle bins (same data set as B). Full distribution of the data values in the grey-shaded areas is shown in Fig. S1, B and B′. **(E)** Mean interface contraction/elongation behavior during area oscillation cycles (oscillation phase angle ϕ), for different interface orientation angle bins (*n* = 1,736 unique interfaces in k = 4 embryos). Full distribution of the data values in the grey-shaded area is shown in Fig. S1, C and C′.

Since there are few cell divisions during the early stages of germband extension (GBE), the majority of the macroscopic tissue lengthening is achieved by simple directional neighbor-exchange intercalation events, referred to as type 1 or t1 transitions. At the cellular level, a t1 process occurs when the interface between two anterior-posterior neighbors (referred to in the following as a “vertical” interface) contracts down to a single higher-order vertex, followed by the creation and elongation of a new interface between the two dorsal-ventral neighbors (referred to in the following as a “horizontal” interface), as shown in Fig. 1 A (Irvine and Wieschaus, 1994; Bertet et al., 2004; Blankenship et al., 2006; Collinet et al., 2015; Yu and Fernandez-Gonzalez, 2017). The t1 transitions pulls the DV cell pair closer together (driving contraction), while pushing the anterior-posterior cell pair farther apart (driving elongation). In addition to t1 transitions, additional contributions to extension are thought to come from cell shape changes, oriented cell divisions, external tissue forces, and from the formation of rosettes (Blankenship et al., 2006; da Silva and Vincent, 2007; Collinet et al., 2015; Butler et al., 2009; Kong et al., 2017); however, our understanding of tissue elongation is far from complete. Over the course of GBE, the germband epithelium elongates by a factor of ∼2.5 (Irvine and Wieschaus, 1994), which would not be achievable via one round of hexagonal-to-hexagonal intercalation alone (Blankenship et al., 2006). While it has been proposed that higher-order rosette structures and cell shape changes throughout development might account for some of this discrepancy, we sought to measure quantitatively whether and how cells participate in multiple t1 transitions to produce higher levels of tissue extension in the early embryo.

The directional preference of t1 transitions, which fundamentally drives the symmetry-breaking of the tissue, requires a system of planar polarity - i.e., the asymmetric localization of certain molecules – within the germband. An important component of this planar polarity is the activation, by anterior-posterior patterning genes, of the RhoGEF2/Rho/RoK pathway at vertical interfaces, where RoK (Rho-Kinase) is the ultimate molecular switch that activates non-muscle Myosin II, which is the primary force-generating/contractile motor protein in the gastrulating embryo (Bertet et al., 2004; Zallen and Wieschaus, 2004; Blankenship et al., 2006; Streichan et al., 2018; Gustafson et al., 2022). Myosin II is preferentially localized to vertical cell interfaces and has been shown to be enriched specifically in contracting interfaces (Bertet et al., 2004; Zallen and Wieschaus, 2004), and it has been proposed that actomyosin networks stabilize line tension in those interfaces to reinforce efficient contraction in intercalating cells (Fernandez-Gonzalez et al., 2009; Rauzi et al., 2008; Kale et al., 2018). In addition, the Bazooka/Par3 scaffolding protein complex, which is associated with cell-cell adhesion, has been shown to polarize within the epithelial sheet with the same temporal onset as Myosin II, but with a complementary localization (Blankenship et al., 2006; Simões et al., 2014). Thus, planar polarity consists of preferential localization of F-actin and Myosin II to vertical interfaces, and preferential localization of E-cadherin and Bazooka to transverse and horizontal interfaces.

However, the degree to which planar polarities may be dynamically reinterpreted is an unresolved question. One mechanistic hypothesis has proposed that planar polarity (of Myosin II and Par-3 localization) is oriented by pair-rule patterning genes. Pair-rule patterning genes (PRGs) are expressed in the embryo in stripes along the AP axis, and the pair-rule model has posited that the primary cue that prompts myosin accumulation at a specific cell-cell contact is a difference in PRG expression levels between the two neighboring cells (Zallen and Wieschaus, 2004; Bertet et al., 2004), with the process likely mediated by Toll-like transmembrane receptors (Paré et al., 2014, 2019; Tetley et al., 2016; Lavalou et al., 2021). This model has also been called the “differential identity model,” because myosin is thought to be concentrated at those interfaces between neighboring cell pairs that differ in their PRG expression identity. Thus, within the framework of this model, Myosin II enrichment should be primarily dependent on the “type” of the interface (homotypic vs heterotypic contact between neighbors), as opposed to the interface’s planar orientation (Tetley et al., 2016). However, several studies have reported that the spatial anisotropy of myosin appears to be robustly dependent on planar angle and is spatially fixed to the DV direction during the first ∼25 min of GBE (Kasza et al., 2014; Tetley et al., 2016; Farrell et al., 2017; Lefebvre et al., 2023).

Another issue is that t1 transitions are typically unidirectional in function, i.e., once a vertical interface has fully contracted and a new horizontal interface has been created, the configuration does not reverse back to a vertical interface (Bertet et al., 2004; Rauzi et al., 2008). Recent work from our group has shown that horizontal interfaces elongate at similar rates as contracting vertical interfaces (Vanderleest et al., 2022); notably, horizontally-oriented interfaces do not stop elongating when they have reached a set length, and even very long horizontal interfaces still show robust - if somewhat slower – elongation behavior (Vanderleest et al., 2018, 2022). Taken together, these data would suggest that a gradual accumulation of very long horizontal interfaces in the tissue should occur over the course of GBE. However, such a systematic accumulation in the tissue has not been observed.

In this work, we report a cellular mechanism that resolves this discrepancy and addresses several unresolved questions concerning interfacial behaviors. We observe that during GBE interfaces systematically rotate toward a vertical orientation angle, and that in doing so, they gradually switch from elongation to contraction behaviors. This mechanism prevents the indefinite elongation of horizontal interfaces, and it explains the absence of an accumulation of extremely long horizontal interfaces. Additionally, this rotation mechanism functions to continuously replenish the population of vertical (and thus contracting) interfaces, thereby increasing the total possible number of interface contractions that a cell can undergo throughout the duration of GBE. As a result, this mechanism has the ability to produce multiple staggered rounds of t1 transitions in each cell, increasing the total achievable macroscopic tissue elongation. Lastly, this mechanism demonstrates that the establishment of planar polarity of the system is not a one-time event that bestows permanent contraction/elongation properties to a specific subset of interfaces, but that planar polarity constitutes a temporally persistent anisotropy of cellular properties, which leads to a continuous reinterpretation and updating of interface behaviors based on their evolving orientation angle.

## Results

### Interface dynamics vary on a continuum with orientation angle

In our previous work (Vanderleest et al., 2022), we demonstrated that newly formed horizontal interfaces in t1 transitions (Fig. 1 A) behave much the same as pre-existing horizontal interfaces, which suggests that horizontal interfaces do not possess a special molecular or behavioral identity that is due to their “history” of originating from a successful t1 transition, but instead that the molecular and/or mechanical properties that act on the interface are directly dependent on the orientation angle. Knowing the boundary conditions that vertical interfaces contract and horizontal interfaces elongate, we wanted to investigate the behavior of the interfaces in the intermediate range of angles—specifically, we wanted to examine whether there was evidence for the existence of distinct dynamic subpopulations, or whether contraction and elongation behavior exists on a continuum.

To answer this question, we first measured the interfaces’ mean rate of length change (contraction/elongation rate, with contraction rate designated as negative and elongation rate as positive), as a function of both interface angle and developmental time (Fig. 1 B, with a sample cell image color-coded for contraction or elongation rate in Fig. 1 B′, see Materials and methods), in a data set comprised of *n* = 2,940 unique interfaces from k = 3 control embryos. Our results indicate that, starting from the onset of GBE, interfaces are robustly contracting in the angle range around θ = 0° (DV aligned) and robustly elongating in the angle range around 90° (AP aligned) as expected. We also see intermediate behavior at intermediate angles, i.e., regions with slower mean contraction or elongation rates and transition regions with net zero rate of change (black arrows in Fig. 1 B). Interestingly, the transition angle (where behavior switches from contraction to elongation) is somewhat variable with developmental time and increases from values θ < 30° at the onset of GBE to θ < 45° over the course of 15 min. These data demonstrate that there are net intermediate behaviors for transverse cell–cell interfaces (i.e., those that are neither strictly vertical nor horizontal).

Next, we wanted to examine whether the observations at intermediate angles constitute a true continuum of behavior, or whether the observed gradient in the mean contraction/elongation rates is the result of a superposition of distinct functional subpopulations (for example, a superposition of one contracting and one elongating subpopulation with variable relative contributions). To examine this possibility, we directly examined the distributions of contraction/elongation rate values at various angle bins (Fig. 1 C), calculated from the same data set as above, but constrained to the time window from 5 to 20 min after the onset of GBE. These distributions have a very significant width (owing to the temporal ratcheting of contraction/elongation behavior of the interfaces), but they appear monomodal, with no evidence of the type of biphasic behavior that we would expect for a superposition of one contracting and one elongating interface population. As a result, we summarized these findings in a box plot showing the contraction/elongation rate change as a function of the interface angle (Fig. 1 C′, all rates are significantly different from each other with P < 0.0001 using an ANOVA analysis followed by Tukey–Kramer test, or as an alternative a Kruskal–Wallis followed by Dunn–Sidak test, see Table S1). It is important to note that the scaling of this rate continuum is time-dependent, and the absolute rates will vary to some degree over the time course of GBE, with maximum contraction rates of vertical interfaces reached ∼5 min after the onset of GBE, while the maximum rate of elongation of horizontal interfaces is not reached until ∼15 min after the onset of GBE (Fig. 1 D). Thus, in summary, we can conclude that at our level of spatiotemporal resolution, cell interfaces contract and elongate along a behavioral continuum with their planar orientation angle.

**Figure S1.**
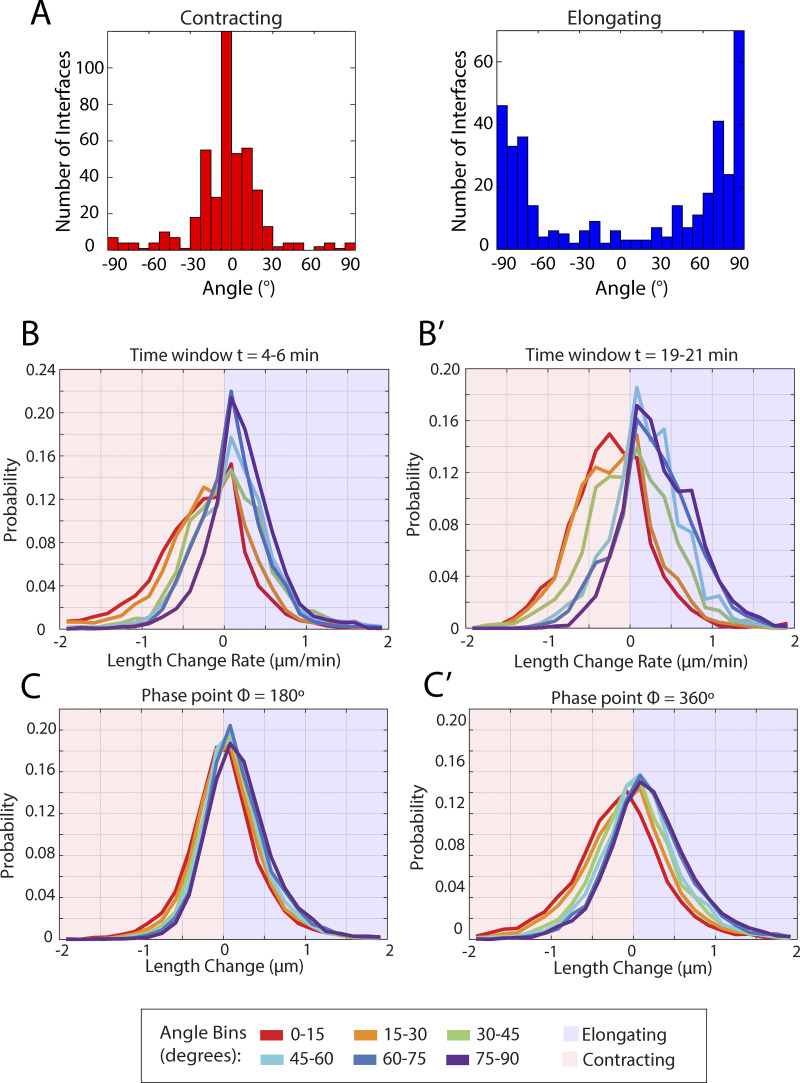
**Contracting versus elongating interface angle measurements and length change data distributions.** **(A)** Histogram of angles for contracting and elongating interfaces during germband extension. (*n* = 449 contracting interfaces, 387 elongating interfaces, k = 3 embryos); **(B)** Distribution of measured interface contraction/elongation rates for different interface orientation angle bins, constrained to the time window t = 4–6 min after initiation of germband extension. (*n* = 2,940 interfaces, k = 3 embryos); **(B′)** Same as B but constrained to the time window t = 19–21 min after initiation of germband extension. **(C)** Distribution of measured interface contraction or elongation rates for different interface orientation angle bins, constrained to the cell area oscillation phase 180° (*n* = 2,940 interfaces, k = 3 embryos). **(C′)** Same as C but constrained to the cell cycle phase 360°.

In addition, we took an orthogonal approach and directly measured the orientation angles of all interfaces that either fully contracted or newly elongated during GBE (Fig. S1 A) in *n* = 836 interfaces in k = 3 control embryos; for best accuracy of the angle measurement in short interfaces, the orientation angle was measured at either the first (elongating interfaces) or the last (contracting interfaces) time point at which the interfaces had a length of 1 μm. The distribution shows that 86.5% of fully contracting interfaces were within ±30° of vertical orientation at their 1 μm timepoint, while 75.4% of elongating interfaces fell within ±30° of horizontal orientation. This result demonstrates that the directional preference is extremely efficient at the level of observable t1 transitions, where horizontal interfaces contribute essentially zero and transverse interfaces only minimally to completed t1 contractions.

**Figure S2.**
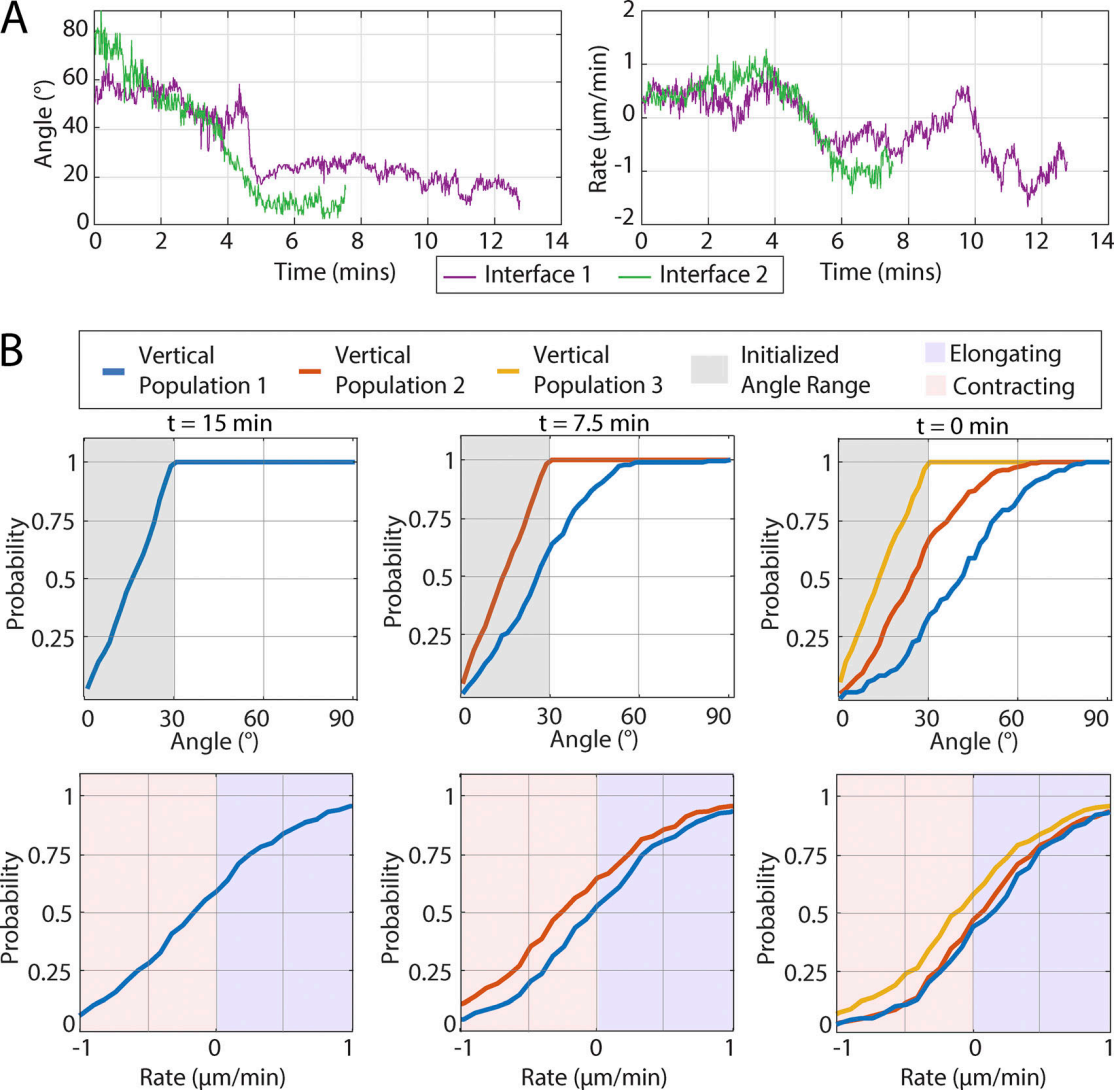
**Rate and angle measurements for example interfaces and the evolution of intially vertical interfaces.** **(A)** Interface angle and interface rate of length change over time shown for the example interfaces given in Fig. 2, A and B. **(B)** Top row: Cumulative probability distribution of interface orientation angles. Bottom row: Cumulative probability distribution of interfaces rate of length change (i.e., contraction/elongation rates). The graphs show the reverse evolution of populations of initially vertical interfaces over a time window of 15 min (*n* = 304 interfaces, k = 6 embryos).

As we had shown previously that vertical contraction and horizontal elongation occur in ratcheted steps in the context of cell area oscillations (Vanderleest et al., 2018), we also wanted to test whether any evidence of ratcheting could be found in transverse interface behavior. When plotting interface length change against area oscillation phase angle ϕ (Fig. 1 E) instead of time t (see Materials and methods), using data from *n* = 1,736 unique interfaces in k = 4 control embryos, we indeed see that transverse interfaces undergo ratcheting along a full continuum of behavior. Comparing vertical to transverse and horizontal interface orientation, the effectiveness of interface contraction during cell area contraction decreases incrementally, and the effectiveness of interface elongation during cell area expansion increases incrementally.

In summary, these data indicate that dynamic interface behaviors occur along an angle-dependent continuum, ranging from maximal contraction at vertical angles to maximal elongation at horizontal angles.

### Individual interfaces change their contraction or elongation behavior as a result of angle rotation

The previous data indicate that, at any given time point during GBE, we can find a range of interface behaviors in the tissue that vary systematically with the interface orientation angle. However, these data do not address how these contraction/elongation behaviors persist over time at the level of the individual interface. For example, it is conceivable that the typical contraction/elongation speed of an interface is initiated once (for example, through the recruitment of a specific amount of myosin) and then persists over time regardless of that interface’s evolving orientation angle. Conversely, it is also possible that contraction/elongation speed is a dynamic property that is set by the interface’s current orientation angle (as opposed to a single initiation event), which means that the behavior should change as the angle changes.

**Figure 2.**
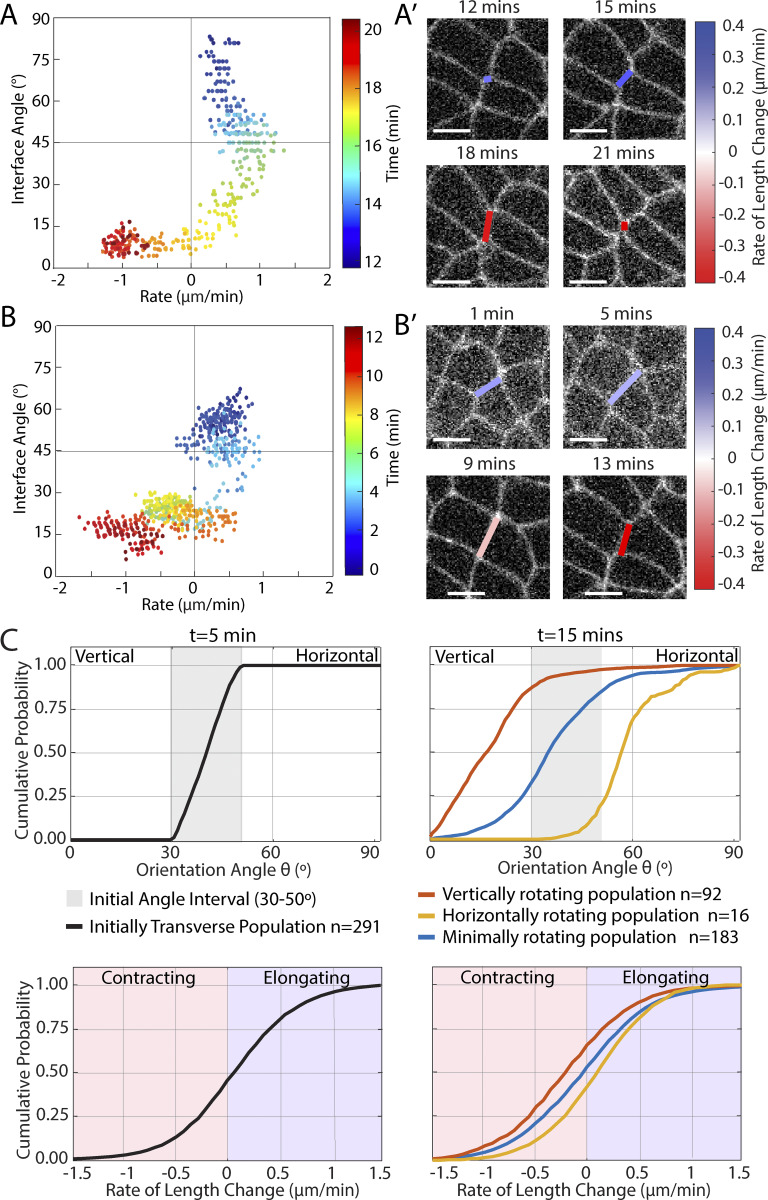
**In individual interfaces, planar polarized behaviors change as the interface rotates. (A and B)** Time evolution of the interface contraction/elongation rate and interface angle in two sample interfaces. The color of the markers indicates time (going from blue to red). **(A′ and B′)** Sample images of interfaces in A and B, color-coded for their measured contraction/elongation rate. (Scale bar = 5 μm) Individual angle as a function of time and rate as a function of time for the sample interfaces is shown in Fig. S2 A. **(C)** Top row: Cumulative probability distribution of interface orientation angles. Bottom row: Cumulative probability distribution of interfaces rate of length change (i.e., contraction/elongation rates). The graphs show the evolution of a population of initially transverse interfaces over a time window of 10 min. The original population is broken down into three subpopulations of interfaces that either stay within ±15° of their original orientation angle or increase or decrease it by ≥15°. (*n* = 291 interfaces, k = 6 movies) Graphs showing the reverse evolution of a population of initially vertical interfaces over a time window of 15 min are shown in Fig. S2 B.

To answer this question, we initially looked into interfaces that undergo a robust change of orientation angle over time. We showed two examples, which demonstrate the evolution of both orientation angle and contraction/elongation rate over time (Fig. 2, A and B, with corresponding sample cell images shown in Fig. 2, A′–B′). The results demonstrate that, in these examples, as the interfaces rotate away from horizontal (AP aligned) to more vertical (DV aligned) orientation, the interface elongation rate decreases and ultimately switches to contraction. This result suggests that contraction/elongation rate is a dynamic behavior that changes during rotation of the orientation angle and shows that interfaces can switch from elongation to contraction behavior over their lifetime.

To verify that this behavior is not only true for these selected samples, but that this is a systematic effect, we identified a cohort of initially transverse interfaces and tracked their orientation and length change rate over time (Fig. 2 C). We started by identifying a “parent” population of interfaces that were transverse during early GBE; specifically, we constrained the data to *n* = 291 unique interfaces from k = 6 control movies, whose orientation angle fell into the range θ = 30–50° at the time point t = 5 ± 2 min, and which were visible in the field of view continuously for at least another 10 min after this initial time point. This original population (black curve) was then divided into three subpopulations based on the interfaces’ rotation behavior during the 10-min time interval: A “minimally rotating” subpopulation of interfaces (*n* = 183) whose orientation angle had remained within ±15° of their starting angle (blue curve); a vertically rotating subpopulation of interfaces (*n* = 92) whose orientation angle had decreased by ≥15° (red curve); and a horizontally rotating subpopulation of interfaces (*n* = 16) whose orientation angle had increased by ≥15° (yellow curve). The top panels in Fig. 2 C show the cumulative probability distribution of interface angles of these populations at the two time points: The parent population at t = 5 min naturally has a sharp angle cutoff. The three subpopulations at t = 15 min are a bit broader, and due to the angle selection criterion, the curve of the vertically rotating subpopulation is of course shifted to the left with respect to the minimally rotating subpopulation, and the curve of the horizontally rotating subpopulation is shifted to the right. The bottom row shows the corresponding cumulative probability distribution of the contraction/elongation rates of these populations. The original population 1 (black curve) is nearly net immobile, with a tiny preference for elongation. But when we examined the three subpopulations 10 min later, we observed that the distribution of the minimally rotating subpopulation (blue curve) shifted very little; the distribution of the vertically rotating subpopulation (red curve) shifted to the left, meaning that its behavior has shifted toward contractile behavior; and the distribution of the horizontally rotating subpopulation (yellow curve) shifted to the right, indicating a shift toward elongation. This result shows that a shift from transverse toward vertical orientation is associated with a systematic shift from elongation toward contraction behavior (and vice versa) and that the contraction/elongation behaviors of interfaces, instead of being predetermined by their orientation angle at the start of GBE, are continuously being “updated” based on the interface’s evolving orientation angle (or more specifically, based on mechanical/molecular properties encoded by the orientation angle).

### Interfaces rotate systematically from horizontal toward vertical orientation

In previous work that specifically studied horizontal interfaces (Vanderleest et al., 2022), we showed that, while newly formed very short horizontal interfaces have the highest elongation speed, longer horizontal interfaces still show robust elongation behavior; in other words, we did not find evidence that horizontal interfaces stop growing at some specific target length. As a result, one would—in theory—expect to see an accumulation of very long horizontal interfaces in the tissue over the course of GBE. However, we didn’t observe this type of accumulation experimentally, and we wondered about this apparent contradiction. The data above suggests that initially horizontal interfaces stop elongating at some point in time not because they reach a threshold length but because they reach a threshold angle, i.e., the interfaces stop growing because they rotate away from their originally horizontal orientation.

Moreover, in the data above, it was notable that the subpopulation of vertically rotating interfaces was much larger than the subpopulation of horizontally rotating interfaces (*n* = 92 rotating toward vertical, as opposed to *n* = 16 rotating toward horizontal orientation), and this discrepancy suggests that interfaces maybe don’t merely “drift away” from their original orientation in a random-walk type fashion, but that there could be a systematic preference for rotation toward the vertical. Thus, the next question we wanted to address was whether horizontal interfaces—or any other interfaces—merely rotate away from their original orientation in a random-walk fashion, or whether there is evidence for systematic changes in the orientation angles.

**Figure 3.**
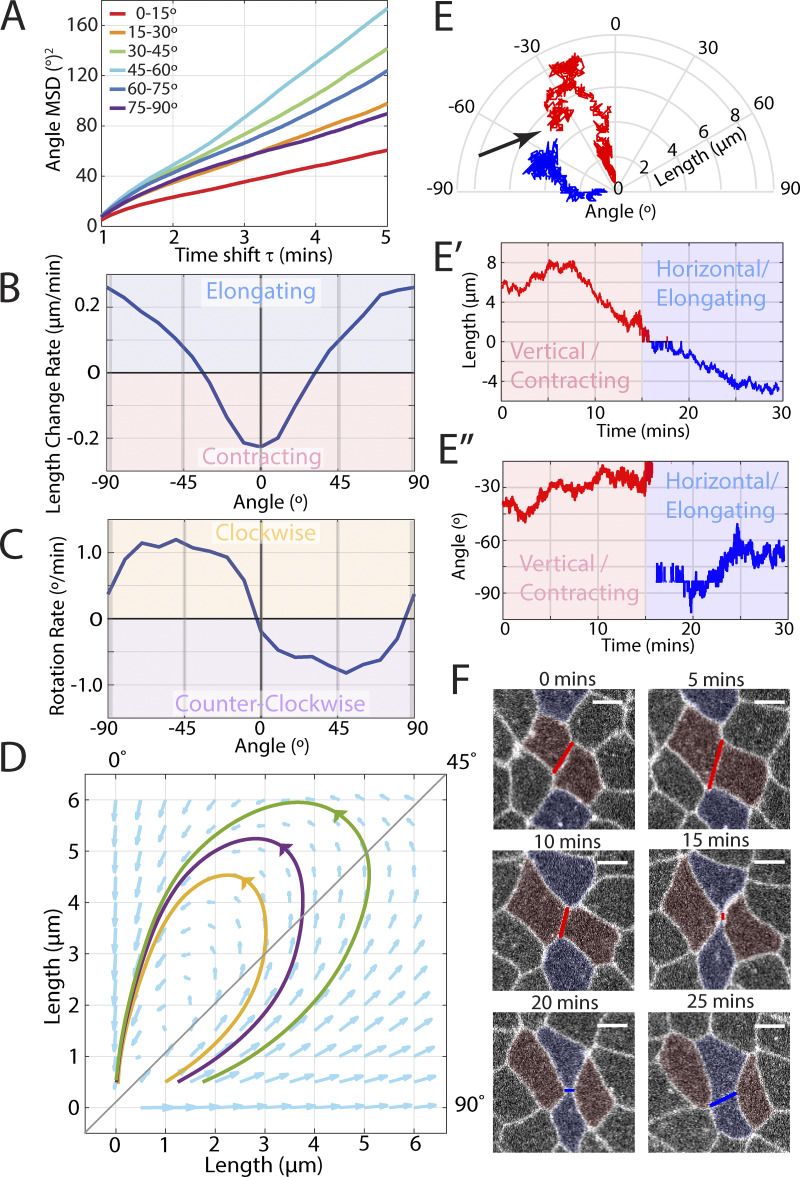
**Systematic rotation of interface orientation angle. (A)** Mean squared displacement of orientation angle change for interfaces with different starting orientations. (*n* = 918 interfaces, k = 3 embryos, see Fig. S3 A for boxplot of distribution of values at each angle range). **(B)** Mean rate of length change over interface orientation angle. (*n* = 5,355 interfaces, k = 7 embryos, see Fig. S3 B for boxplot of distribution of values in grey shaded areas). **(C)** Mean rate of rotation over interface orientation angle. (*n* = 5,355 interfaces, k = 7 embryos, see Fig. S3 C for boxplot of distribution of values in grey shaded areas). **(D)** Polar quiver plot indicating local direction of angle and length change, with streamlines overlaid. (*n* = 5,355 interfaces, k = 7 embryos). **(E)** Polar plot of a sample t1 transition with contracting interface in red and elongating interface in blue. (black arrow denotes starting time of t1 transition). **(E′)** Interface length over time for the same sample as above. **(E″)** Interface angle over time for the same sample as above. **(F)** Raw images of the same sample as above with false-color overlay of cells and interfaces. (Scale bars = 5 µm).

To examine this question, we separated interfaces into angle bins of 15° based on their average orientation angle during the first 5 min of GBE and calculated the mean squared angular displacements (MSD) for their trajectories (Fig. 3 A). Surprisingly, the results show that the interface populations close to vertical or horizontal orientation have the lowest net angular displacements (and thus the lowest net rotation tendency), while the “transverse” interfaces have the most robust angular displacements. Moreover, these transverse interfaces also show clear evidence of “upward curvature” in the MSD, indicating that the trajectories have a degree of super-diffusive behavior (with a fitted exponent γ > 1 as shown Table S2). This means that transverse interfaces do indeed rotate systematically, as opposed to only undergoing random-walk type orientation changes.

**Figure S3.**
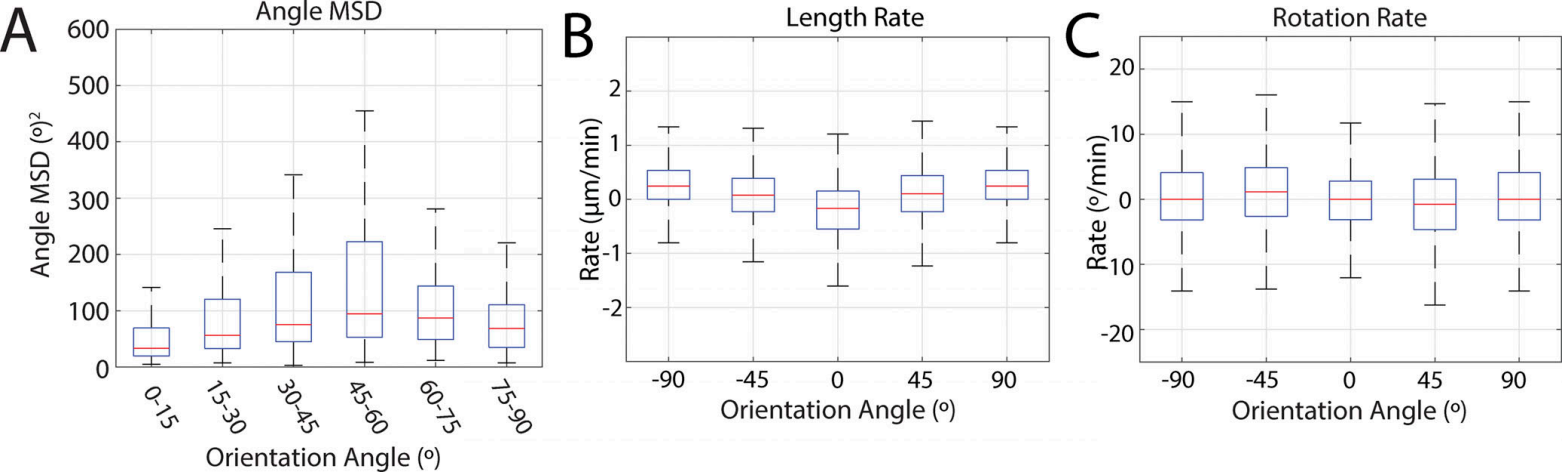
**Boxplots for interface angle MSD, length rate, and rotation rate distributions.** **(A)** Box plot of the mean squared displacement values for different interface orientation angle bins given in Fig. 3 A. (*n* = 918 interfaces, k = 3 embryos); **(B)** Box plot of length rate change for different orientation angles denoted in Fig. 3 B. (*n* = 5,355 interfaces, k = 7 embryos); **(C)** Box plot of rotation rate for different orientation angles denoted in Fig. 3 C. (*n* = 5,355 interfaces, k = 7 embryos).

As a result, we examined the systematic rotation component directly for a range of interface angles (Fig. 3, B and C). For comparison, we measured the angle-dependent mean length change rate (contraction/elongation rate), and the corresponding angle-dependent mean angle change rate (rotation rate) (for error/statistics of these data curves, please see Fig. S3). We find that the length change rate is positive when the interfaces are horizontal (±90°) and negative when the interfaces are vertical (0°), reaching zero around ±35° (Fig. 3 B), consistent with our above results in Fig. 1 B. The corresponding rotation rate goes to zero both at horizontal and vertical interface orientation, reaching its (positive) maximum close to the diagonal −45° and (negative) minimum around +45° (Fig. 3 C). Conceptually, this means that negative orientation angles tend to increase (i.e., moving the orientation toward zero, which equals vertical) and positive orientation angles tend to decrease (i.e., also moving the orientation toward zero, which equals vertical). This means that interfaces can rotate both in clockwise and counterclockwise directions, but the common principle is that they systematically rotate into whichever direction brings them closer to vertical orientation, with the highest net rotation speed observed for transverse interfaces.

We demonstrate this directional preference in a timelapse movie (Video 1), which shows a sample cell with color-coded interfaces, and a plot of the evolving angular orientation of all of its interfaces. The cell participates in multiple t1 transitions so that it has several disappearing vertical and newly created horizontal interfaces. The plot shows that the cell’s interfaces can rotate both clockwise and counterclockwise, but that all of the angle trajectories, albeit noisy, converge toward vertical orientation (0°).

**Video 1.** **Cell interfaces preferentially rotate toward vertical: Video of a single cell (shaded blue) over time with the cell interfaces overlaid in different colors.** The plot on the right shows the angle of each interface as a function of time, with the color of the plotted interface corresponding to the interface color overlaid in the cell video. Playback speed: 10 frames per second.

In addition, we illustrated the combined effect of length and angle changes in a polar plot of a sample trajectory of a t1 transition (Fig. 3 E). The contracting interface (red) starts in a transverse orientation with a relatively long length; and as the angle rotates closer to vertical, the interface contracts into a higher-order vertex. The vertex resolves into a new horizontal interface (blue) and initially rapidly elongates, but its elongation speed decreases as the interface rotates toward a transverse orientation (Fig. 3, E′ and E″, corresponding live cell image are shown in Fig. 3 F).

These data show that both length contraction/elongation and rotation speed are dependent on the current orientation angle; in addition, we know from previous work (Vanderleest et al., 2022) that contraction/elongation rates also have a length-dependent contribution. Thus, since we know that length and angle interact, we wanted to visualize the combined effects of systematic length and angle changes for the full “phase space” of typical interface length and orientation angle values. For this purpose, we interpolated the instantaneous length and angle change rates during GBE across all angles and across a range of 0–6 μm from a large set of experimental data (*n* = 5,355 interfaces, k = 7 embryos) and plotted one quadrant of the results as a quiver plot in a polar coordinate system (Fig. 3 D). Conceptually, for any point in this plot, the radial distance from the origin represents interface length, and the circumferential angle represents the interface orientation angle θ. The quiver vectors represent the direction of instantaneous change: Vector components pointing radially outward or inward to the origin indicate an increase or decrease in interface length, while vector components pointing tangentially to the origin indicate a change in interface orientation angle. In addition, we have added “streamlines” to the quiver plot, which represent physically feasible continuous trajectories of interfaces through the parameter space in time. Videos 2 and 3 show two examples of these physically feasible trajectories with time resolution, and they represent the “typical” time-resolved behavior of two interfaces (one with clockwise, the other with counterclockwise rotation) in an analogous four-quadrant plot, where the midpoint of the interface is fixed to the origin and the endpoints of the interfaces follow the streamlines.

**Video 2.** **Example of typical combined interface rotation and elongation/contraction behavior: Polar quiver plot indicating the local direction of angle change and length change, with sample streamlines overlaid.** (Interpolated from *n* = 5,355 interfaces, k = 7 embryos, as shown in Fig. 3 D). The newly formed horizontal interface (shown in black), after breaking symmetry into counter-clockwise direction begins rotating toward a vertical orientation angle, following the yellow streamline. Playback speed: 60 frames per second.

**Video 3.** **Example of typical combined interface rotation and elongation/contraction behavior: Polar quiver plot indicating local direction of angle change and length change, with sample streamlines overlaid.** (Interpolated from *n* = 5,355 interfaces, k = 7 embryos, as shown in Fig. 3 D). The newly formed horizontal interface (shown in black), after breaking symmetry into clockwise direction, begins rotating toward a vertical orientation angle, following the purple streamline. Playback speed: 60 frames per second.

These results reproduce our finding above that horizontal interfaces primarily elongate (with little to no systematic rotation), that vertical interfaces primarily contract (with little to no systematic rotation), and that transverse interfaces primarily rotate (with little to no systematic contraction/elongation), with a continuum of intermediate behavior at intermediate angles. Interestingly, our data also show that the parametric streamlines are not perfectly symmetric around the diagonal; peak rotation speed occurs not exactly at 45° but toward smaller angles around ∼35°. Taken together with the MSD results from Fig. 3 A, these results suggest that horizontal interfaces have no established preference yet for either clockwise or anticlockwise rotation, but that they initially drift away from their original horizontal orientation through a random-walk type process. However, analogously to an unstable equilibrium, as soon as these interfaces gain any meaningful net rotation from this random process, they also establish a preferential rotation direction, which always directs them further away from the horizontal and causes them to break symmetry. As interfaces rotate away from the horizontal orientation, their elongation slows, then ultimately stalls as they become transverse, and finally reverses to contraction behavior as they become increasingly vertical. Robustly vertical interfaces do not have the opportunity to rotate much further because they contract rapidly and disappear into a higher-order vertex. In summary, these data show that interfaces undergo a systematic rotation from the horizontal toward the vertical, i.e., a directional “rectification,” throughout the germband process.

### Disrupting contractile molecular machinery disrupts rotation

**Figure S4.**
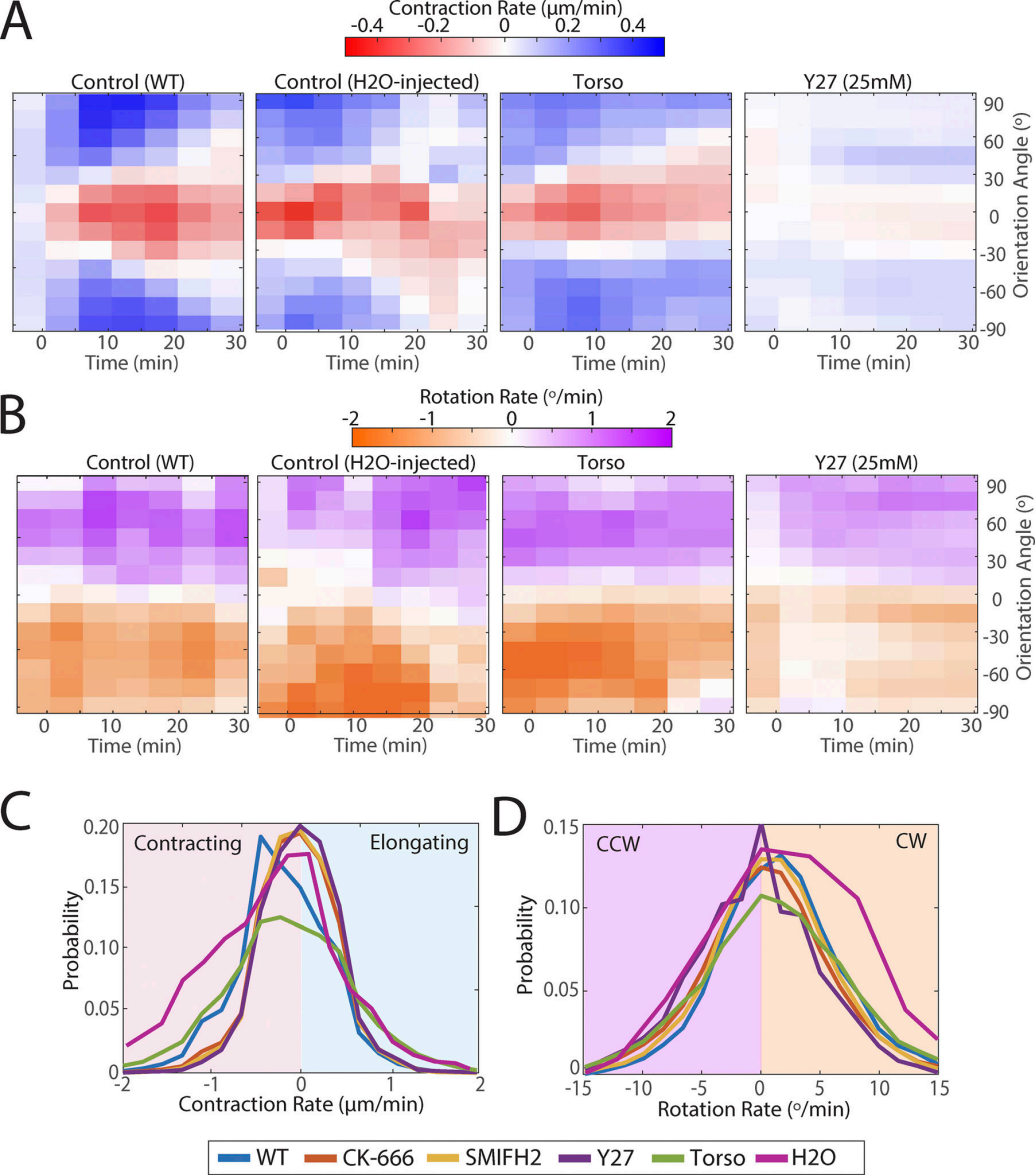
**Effects of Rho kinase inhibition and Torso disruption.** **(A)** Heatmaps of the rate of change in interface length (red: contraction, blue: elongation) as a function of time and interface orientation angle for movies of small-molecule-treated embryos, specifically 25 mM Y27632 (Rho kinase inhibition, disrupts all pools of myosin) (*n* = 1,477 interfaces, k = 3 embryos), as well as genetic disruptions, including KOs of Torso (disrupts posterior midgut formation) (*n* = 2,665 interfaces, k = 5 embryos). The appropriate control for Y27 injection is “H_2_O injected,” while the appropriate control for Torso KO is “WT.” **(B)** Heat map of the rotation rate (orange: clockwise, purple: counterclockwise) as a function of time and interface angle for the same data sets as in A. **(C)** Probability distributions of contraction/elongation rates of the treatments in Figs. 4 and S4. **(D)** Probability distribution of rotation rates of the treatments in Figs. 4 and S4.

**Figure 4.**
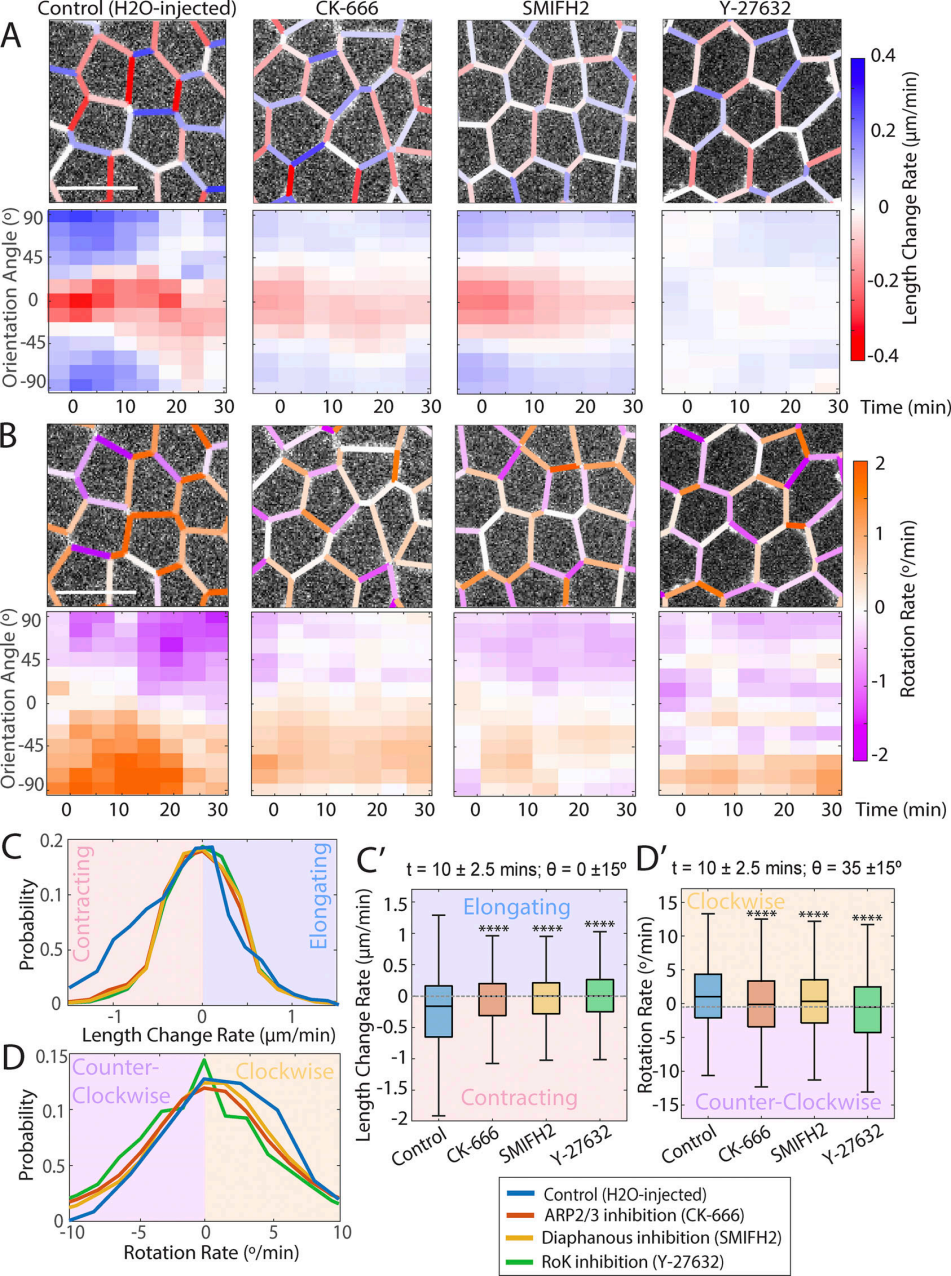
**Reduced force generation leads to changes in angle rotation rate. (A)** Heatmaps of the rate of change in interface length (red: contraction, blue: elongation) as a function of time and interface orientation angle for movies of control and small-molecule-treated embryos, specifically H_2_O-injected Control (*n* = 2,534 interfaces, k = 3 embryos), CK-666 (*Arp-Inhibition*) (*n* = 2,321 interfaces, k = 4 embryos), *SMIFH2* (*Diaphanous-Formin Inhibition*) (*n* = 2,306, k = 4 embryos), and 100 mM Y27632 (*Rho kinase inhibition*) (*n* = 1,240 interfaces, k = 3 embryos). Associated examples of real-cell images taken at ∼5 min after the onset of germband extension, with interfaces false-colored based on instantaneous contraction/elongation rate. Scale bars = 10 μm. **(B)** Heat map of the rotation rate (orange: clockwise, purple: counterclockwise) as a function of time and interface angle for the same data sets as in A. The same real-cell images are false-colored based on instantaneous rotation rate. Scale bars = 10 μm. **(C and C′)** Probability distributions of contraction/elongation rates of the same treatments; (C′) corresponding box and whisker plots (C′). **(D and D′)** Probability distribution of rotation rates of the same treatments; (D′) associated box and whisker plots. Statistical significance was determined with two-sample Kolmogorov–Smirnov test (****P < 0.0001).

We hypothesize that interface rotation is driven by a combination of molecular and mechanical factors; it is conceivable that the contraction of a vertical interface per se drives the rotation of neighboring transverse interfaces, by pulling them into a more vertical geometry. To test this idea, we perturbed the function of known force-generating molecules. Specifically, we perturbed Myosin II, the motor protein required for interface contraction, by inhibiting the upstream Rho kinase RoK (using 25 and 100 mM Y27632, see also Fig. S4). We also disrupted the actin cytoskeleton since interactions between actin and myosin act as the primary force-generating mechanism in our system through inhibitions of the ARP2/3 (250 μM CK-666) and Diaphanous/Formin complexes (50 μM SMIFH2), the major actin nucleating factors. Contraction/elongation and rotation rates were plotted over time and angle as heatmaps for the disrupted embryos (Fig. 4, A and B). Inhibiting these contractile machinery showed significant reductions in contraction/elongation rates, as expected. Further, these disruptions showed concurrent reductions in interface rotation rates, supporting a hypothesis that contracting interfaces play an active role in driving a rotation mechanism. Characteristic cell images depicting these reduced contraction/elongation rates and rotation rates are shown next to their associated heatmaps, each showing the timepoint 5 min after the onset of GBE.

In addition, we also sought to probe potential larger-scale tissue forces that could influence interface rotation. To do this, we analyzed Torso mutant embryos, in which the formation of the posterior midgut is disrupted. The posterior midgut applies an anterior–posterior–axis–aligned pulling force that has been shown to assist in interface alignment throughout GBE (Collinet et al., 2015). These embryos did not show significant differences in control embryos, either in contraction/elongation or rotation rates (Fig. S4). This could indicate that rotation is largely driven by local forces at the individual interface level as opposed to larger-scale tissue forces.

Systematic quantification of the effect of Actomyosin disruptions was performed for the time window 7–13 min after the start of GBE (Fig. 4, C–D′) because contraction/elongation speed peaks around the 10-min mark after the onset of GBE (Vanderleest et al., 2022); length change rates were measured in vertical interfaces (θ = 0 ± 15°), which have the most robust contraction rates. Rotation rates at this time point were measured in transverse interfaces that fall within the range θ = 35 ± 15°, corresponding to the angle interval with the peak rotation speed. While all distributions are fairly broad, a significant shift is seen between control and the different actomyosin disruptions, with severe defects of both contraction and rotation in the disrupted embryos. Our results show that actomyosin force generation is, at least in part, required for systematic interface rotation to occur.

### Interfaces show an angle-dependent gradient of junctional myosin intensity

At the molecular level, symmetry breaking of t1 transitions is achieved through a system of planar polarity, consisting of asymmetrical molecular populations of F-actin and Myosin II (preferentially located to vertical interfaces), complementary with a population of Ecadherin and Bazooka (preferentially located to transverse/horizontal interfaces) (Bertet et al., 2004; Zallen and Wieschaus, 2004; Blankenship et al., 2006), and it has been established that the selective contraction of vertical interfaces requires myosin function (Fernandez-Gonzalez et al., 2009). Our results above suggest that planar polarized behavior isn’t a permanent property that is bestowed upon one set of interfaces at one single time point, but that rotation allows interfaces to acquire both vertical orientation and the ability to contract on an ongoing basis. As a result, we wanted to examine whether these rotating interfaces also acquire myosin dynamically over time by tracking the myosin intensities of dynamic interfaces (see Materials and methods for details).

**Figure 5.**
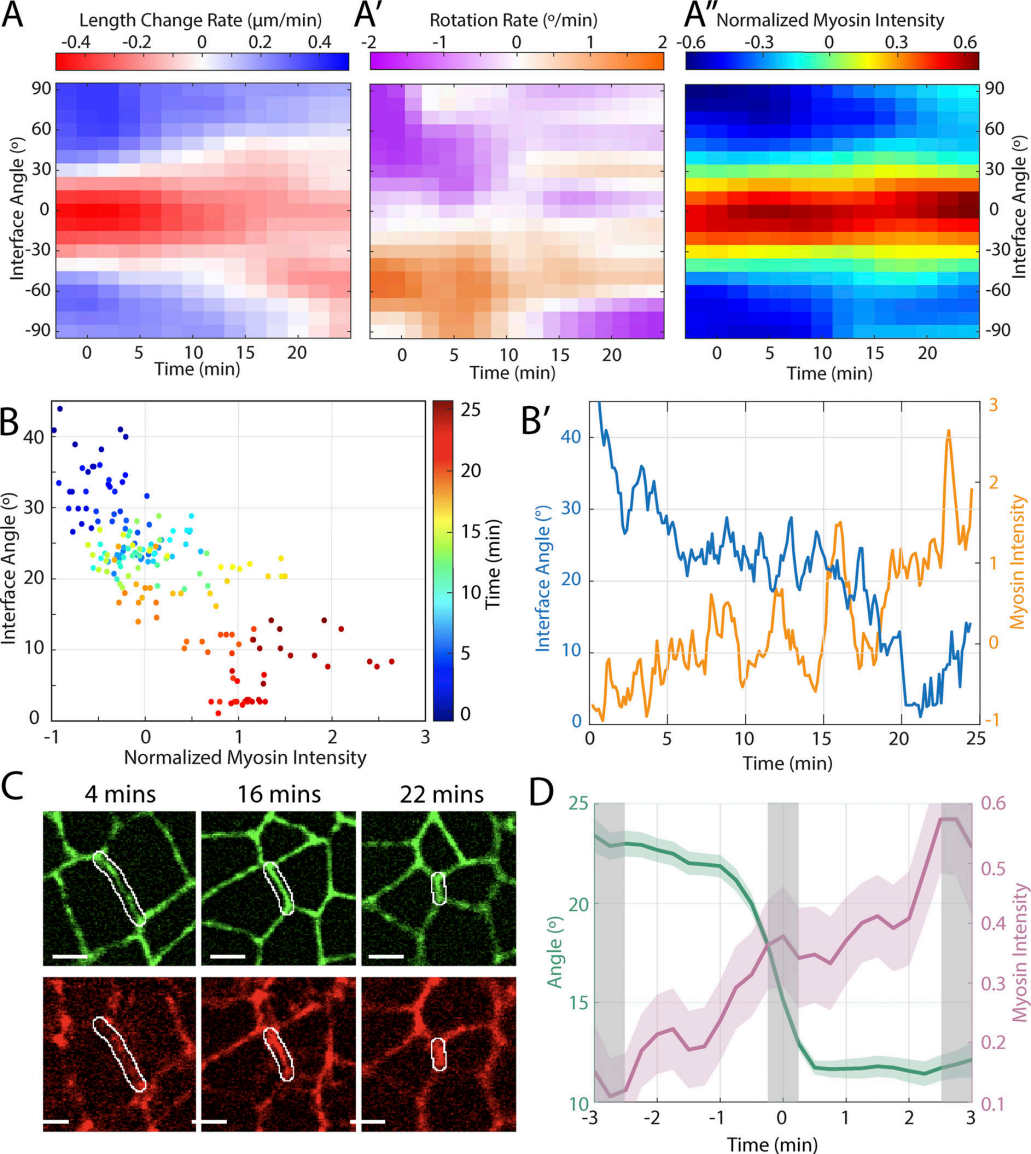
**Junctional myosin in interfaces is dependent on angle.** **(A)** Heat map of the rate of change in interface length (red: contraction; blue: elongation) as a function of time and interface orientation angle. (*n* = 1,210 interfaces, k = 3 embryos). **(A′)** Heat map of the rotation rate (orange: clockwise, purple: counterclockwise) as a function of time and interface orientation angle, for same data set as A. **(A″)** Heat map of normalized myosin intensity (red: positive intensity; blue: negative intensity) as a function of time and interface orientation angle, for same data set as A. **(B)** Time evolution of the interface myosin intensity and interface angle in a sample interface. The color of the markers indicates time (going from blue to red). **(B′)** Interface myosin intensity and angle as a function of time for the same sample interface. **(C)** Raw microscope images of the same sample interface at 4, 16 and 22 min into germband extension in E-cadherin and Myosin channels. (Scale bars = 5 µm) An additional interface example is given in Fig. S5, C, C′, and D with the same graphs and images as in Fig. 5, B, B′, and C. **(D)** Orientation angle and myosin intensity evolution in transverse interfaces. (*n* = 87 interfaces, k = 3 embryos) See Fig. S5 B for boxplot of the distribution of values in grey shaded areas.

**Figure S5.**
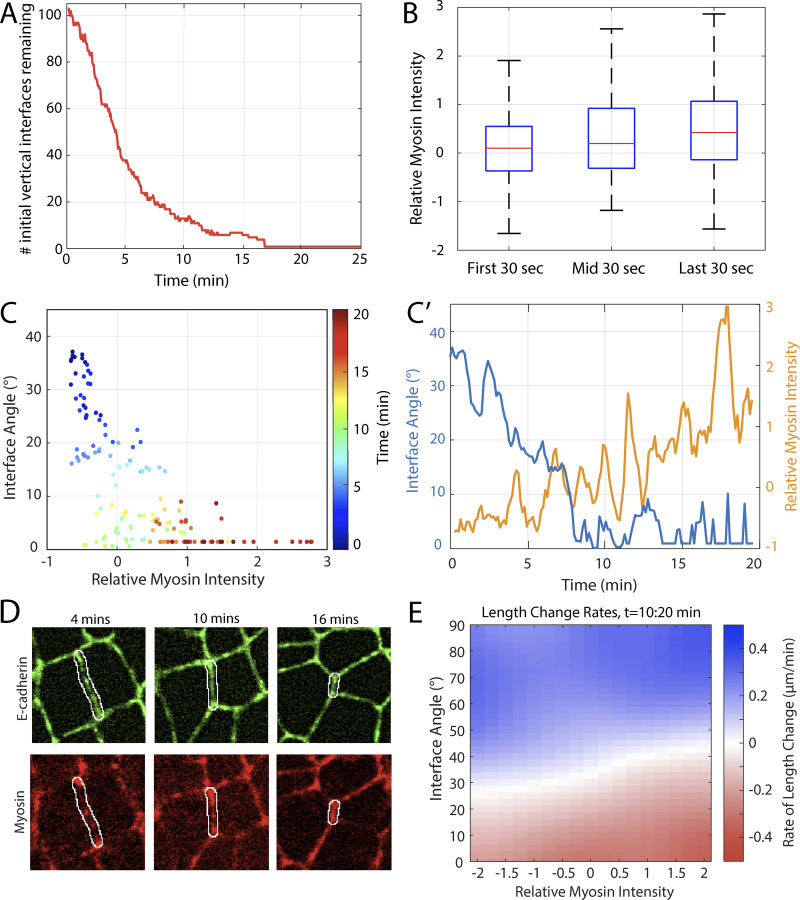
**Expanded myosin contractility analyses.** **(A)** Number of initially vertical interfaces (within ± 15° of vertical) remaining over time in germband extension (*n* = 129 interfaces, k = 6 embryos). **(B)** Box plot of the relative myosin intensity for three different time periods denoted in Fig. 5 D. **(C)** Time evolution of the interface myosin intensity and interface angle in a sample interface. The color of the markers indicates time (going from blue to red). **(C′)** Interface myosin intensity and angle as a function of time for the same sample interface. **(D)** Raw microscope images of the same sample interface at 4, 10 and 16 min into germband extension in E-cadherin and Myosin channels. (Scale bars = 5 μm). **(E)** Heat map of the rate of change in interface length (red: contraction; blue: elongation) interpolated as a function of interface orientation angle and relative myosin intensity. (*n* = 2,296 interfaces, k = 6 embryos).

If there were only a single significant myosin initiation event at the onset of GBE, then we would expect the relative intensity of myosin to go from a strong planar polarized distribution at the beginning of GBE to a more isotropic distribution of intensities across all angles at a later time point, reflecting the fact the initial population of myosin-rich vertical interfaces disappears over time through contraction. However, our analysis of angle-dependent myosin intensities (Fig. 5 A″, heatmaps of length change rate and rotation rate shown as reference in Fig. 5, A and A′) shows a strong planar polarized myosin population—a band of high myosin intensity associated with vertical interfaces—sustained robustly over the entire course of GBE, i.e., over the entire time period in which robust planar polarized contraction/elongation behavior and angle rotation persists. Importantly, this is in spite of the fact that the population of initially vertical interfaces (i.e., interfaces that are vertical within ±15° at the start of GBE) decays to half of its initial number within just the first 5 min (Fig. S5 A). This temporal persistence of planar myosin polarity, despite the swift loss of the initial population, indicates that there must be a mechanism to replace the disappearing vertical interfaces by an equally myosin-enriched new population. This suggests that, as transverse interfaces undergo rotation toward the vertical, they also acquire myosin.

We tested this hypothesis directly by measuring the relative change in myosin intensity that is associated with a decrease in interface orientation angle (i.e., rotation toward vertical). The trajectory of a sample interface (angle orientation vs myosin intensity in Fig. 5 B, with individual time plots in Fig. 5 B′) shows that rotation toward the vertical is associated with myosin enrichment; this is also reflected in the corresponding cell images in myosin channels (Fig. 5 C). To determine whether this is a systematic effect, we repeated this analysis in a larger population of rotating interfaces (Fig. 5 D). Based on the myosin heatmap in Fig. 5 A, we expected myosin recruitment to occur in the angle range below 30°, so we constrained our data to a set of vertically rotating interfaces whose orientation angle decreased to below 10°. We aligned the original time courses of these interfaces to a reference time point (defined as the median time point between the last time point when the orientation angle was ≥20° and the first time point where the orientation angle was ≤10°) and further constrained the analysis to those interfaces that were continuously visible from ±3 min before after this reference time point, yielding *n* = 87 interfaces from k = 3 control embryos. This analysis confirms that a decrease in the orientation angle is associated with a systematic increase in myosin intensity, demonstrating that interfaces systematically recruit myosin as they rotate toward the vertical. In addition, within this data set, the interfaces with relatively higher myosin intensities had a higher tendency toward contraction than the interfaces with lower myosin intensities of the identical angle (Fig. S5 E). Thus, taken together, these results show that transverse interfaces rotate toward vertical orientation, and in doing so acquire both myosin and the ability to contract on an ongoing basis throughout the course of GBE.

### Cells undergo multiple rounds of t1 transitions during germband elongation

**Figure 6.**
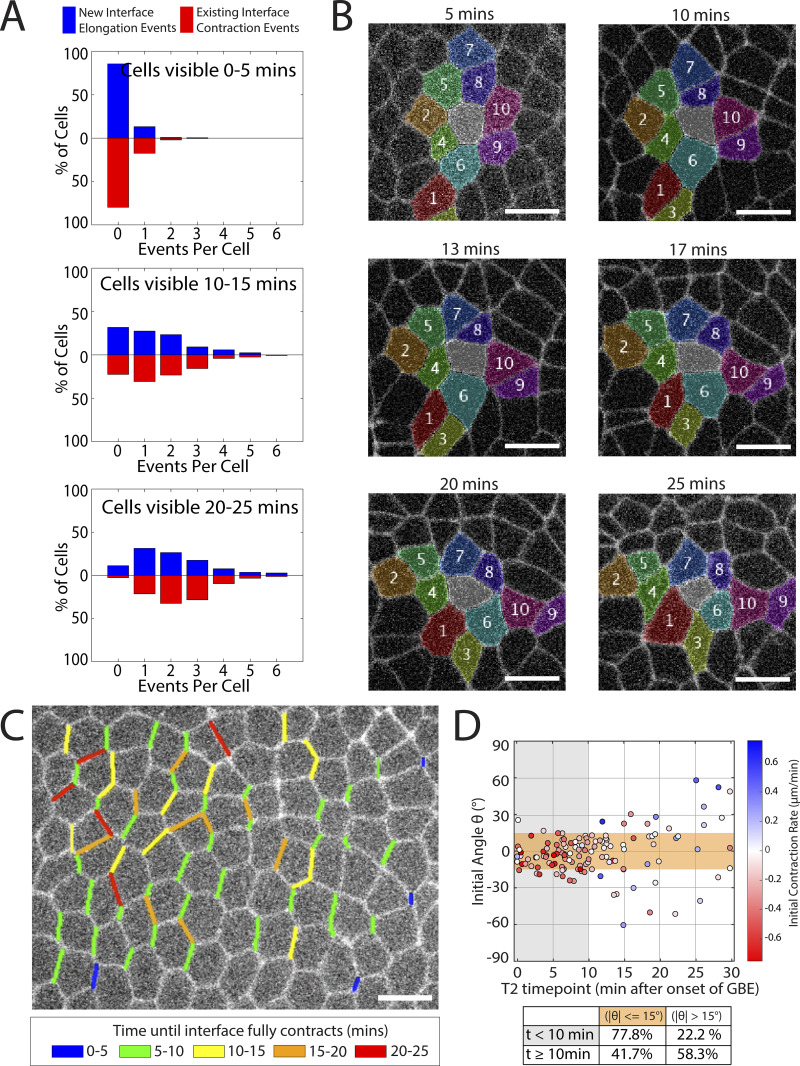
**Cell interfaces change behavior, leading to multiple rounds of intercalation. (A)** Counts of interface changes over time. Each subgraph represents a time period that cells are visible during germband extension (red: contraction; blue: elongation). (*n* = 726 cells, k = 3 embryos). **(B)** Sample images at different time points of a cell that has five contracting interfaces and three elongating interfaces. Scale bars = 5 µm. **(C)** Still image of the start of germband extension with contracting interfaces highlighted to indicate the time period until the interface contracts. **(D)** Initial interface orientation angle versus timepoint of the higher-order vertex configuration, color-coded by initial contraction/elongation rates (*n* = 129 interfaces, k = 6 embryos).

Many mental models for t1 transitions in germband intercalation, which are reflected in the model diagrams we use to illustrate the process or in the explicit rules for computational models of the t1 process (e.g., Rauzi et al., 2008; Siang et al., 2018), assume that contractile behavior is a permanent property of a vertical interface over certain timescales. In computational models, this assumption actually causes the planar polarity of the tissue to disappear when all the vertical interfaces have successfully contracted so that this model produces a single round of intercalation. Experimentally, this “single round” model would cause each cell to typically have about two interfaces that are sufficiently vertical to undergo t1 transitions. However, our data above suggests that transverse interfaces can rotate into the vertical angle range—and concurrently acquire contractile behaviors—many minutes after the initialization of planar polarity and the onset of GBE, which suggests that planar polarity is a persistent asymmetry of behavior that is continuously reinterpreted based on spatial geometry. Together with the observed systematic rotation, this suggests that cells could potentially undergo additional t1 transitions (i.e., more than the predicted two per cell) over time, through the ongoing recruitment of originally transverse interfaces into the vertical contracting interface population. We tested this hypothesis by measuring directly how many topological transitions (either interface disappearances through a full contraction or appearance of the new interface through the resolution of higher-order vertices) occurred per cell over time during GBE (Fig. 6 A) (*n* = 726 cells, k = 3 embryos). Since many cells move in or out of the field of view during imaging, we grouped cells by how long they were continuously visible during GBE. These data show that, for cells that remain in the field of view for longer durations, we can also observe more t1 transitions: We typically observe between zero and one t1 transitions in cells that are visible for <5 min, and typically about two t1 transitions in cells that are visible 10–15 min. However, in cells that are visible >20 min, it is common to see three to four transitions, including some cases of five or six transitions in a single cell (see Table S4). The fact that the number of observed t1 transitions increases with observation time is consistent with the idea that cells undergo t1 transitions continuously, where the pool of contractile interfaces is continuously “replenished” through the rotation of transverse interfaces toward the vertical.

As this statistical data is aggregated from multiple movies, we wanted to examine what this behavior looks like at the level of an individual cell. For this purpose, we showed an example of multiple t1 transitions occurring in a cell that is visible continuously for 25 min over the course of GBE (Fig. 6 B). Over this observation time, the center cell (colored grey in the image) undergoes a total of five interface contractions, which causes it to lose contact with some of its initial neighbors (cells 2, 4, 5, 9, and 10), and three interface elongations, where it gains contact with new neighbors (cells 1, 3, and 7). Despite multiple interface contractions, the cell does not form an obvious rosette structure since the contractions are staggered sufficiently in time.

To visualize this effect at the level of the tissue, we produced a still image from the start point of GBE, where interfaces are color-coded for the length of time after which the interface will fully contract (Fig. 6 C, also see Video 4). Consistent with our data above, interfaces that fully contract within 5–10 min of the start of GBE (blue and green) tend to be already well aligned with the vertical direction, while initially transverse interfaces don’t fully contract until later in the movie since they require more time to rotate before acquiring contractile ability. Within the image shown in the figure, the cells in the bottom region will move out of the field of view within a few minutes, while the top region stays visible for longer. Within the region with long visibility, there are multiple cells that have three, four, or sometimes even five interfaces that will eventually contract at some point during GBE.

**Video 4.** **Initially non-vertical interfaces contract after rotating: Video of a section of germband tissue.** Interfaces are color-coded for the length of time after which the interface will fully contract. Blue: Contract within 5 min; Green: Contract within 10 min; Yellow: Contract within 15 min; Orange: Contract within 20 min; Red: Contract within 25 min. Playback speed: 7.5 frames per second.

Together, these data show that t1 transitions are happening continuously over the course of GBE through the continuous reinitialization of contraction behavior in interfaces that rotate from transverse to vertical orientation. As a result, it is not uncommon for cells to have more than two contracting and two elongating interfaces over the course of GBE, which suggests that this behavior can produce higher effective convergent extension.

To quantify how much these additional t1 transitions originating from rotating interfaces contribute to cell intercalation (and therefore tissue elongation), we identified a set of *n* = 129 interfaces in k = 6 embryos that are visible continuously from the start of GBE at t = 0 and that are seen to undergo a full t1 transition—i.e., we observed both the formation and resolution of a higher-order vertex. We measured their “starting angle” (i.e., the orientation angle of the interface in question at the start of GBE at t = 0) and the developmental timepoint t at which the interfaces reach the midpoint of the t1 transition (i.e., the timepoint representing the higher-order vertex configuration) (Fig. 6 D). It is important to note that many cells move out of the field of view over time so that the number of cells that are continuously visible from t = 0 decreases with the length of the time interval—thus, the sparse sampling for larger time values reflects observation bias and not an inherently reduced frequency of t1 events with time. We see that those interfaces that undergo successful t1 transitions within the first 10 min of GBE typically have starting angles of within ±30°, meaning that these interfaces were already vertical or close to vertical at the beginning of GBE. However, interfaces that undergo transitions later than 10 min into GBE tend to have significantly larger starting angles, consistent with the rotation behavior we describe above: These interfaces were often closer to transverse orientation at the beginning of GBE (30–45°) and also include some interfaces with starting angles around 60°—meaning that these interfaces were more horizontal than vertical and that they would not have been identified as candidates for future t1 transitions at the start of GBE based on their orientation angle alone.

Thus, the t1 transitions that occur within the first ∼10 min of GBE represent the population of interfaces that were already vertical at the start point of GBE, consistent with canonical models of planar polarity initiation and intercalation. However, the t1 transitions that occur >10 min after the start of GBE increasingly originate from interfaces that were originally transverse or horizontal at t = 0, and which achieve successful contraction only after rotating toward a more vertical orientation angle. Thus, these t1 transitions represent “bonus” transitions (of initially non-vertical interfaces), which have the macroscopic effect of increasing the net tissue intercalation beyond the value that the canonical process—a single round of intercalation of vertical interfaces initiated at t = 0 – would be able to achieve.

## Discussion

It has long been known that the necessary spatial symmetry-breaking for directional intercalation in the germband comes from the preferential contraction of vertical (dorsal-ventral oriented) and preferential elongation of horizontal (anterior-posterior oriented) interfaces (Irvine and Wieschaus, 1994; Bertet et al., 2004; Blankenship et al., 2006; Collinet et al., 2015; Yu and Fernandez-Gonzalez, 2017). Interestingly, however, here we find no evidence for separate and distinct populations of interfaces (e.g., one contracting vertical, one elongating horizontal, and one inert transverse population). Instead, our results show that the contraction/elongation behavior varies on a continuum with orientation angle, where interfaces of intermediate angles show intermediate behavior, including slow or no contraction/elongation. Intriguingly, the switch angle (i.e., the orientation angle corresponding to the switch from contracting to elongating behavior) varies with time throughout GBE. In addition, these behaviors all occur in the ratchet-like framework of cell area oscillations, in which a gradual increase/decrease in the directional preference of the ratchet produces a gradual increase/decrease of the net contraction or elongation speed of the interface.

With this behavioral gradient in mind, we wanted to determine if an interface’s contraction/elongation behavior is a fixed or dynamic property—in other words, whether the behavior is initialized once at the beginning of GBE, after which it persists over time regardless of any subsequent changes in orientation angle, or whether the behavior of a given interface changes when its orientation angle changes. Our results clearly indicate that contraction/elongation behaviors are dynamic, where individual interfaces increase or decrease their contraction/elongation speed when they change angle, and where a given interface can even switch its behavior from robustly elongating to robustly contracting, as long as it rotates sufficiently during its lifetime.

Interestingly, we also find that substantially more transverse interfaces rotate toward the vertical direction than toward the horizontal. A quantitative analysis of rotation direction confirmed that this tendency is indeed a systematic preference for rotation of interfaces from horizontal toward vertical orientation—an angle rectification. This rotation preference is strongest for transverse interfaces and weakest for vertical and horizontal. However, an originally horizontal (elongating) interface can gain meaningful rotation over time through random-walk type orientation changes, and once it has random-walked away sufficiently from horizontal orientation in either direction, it breaks symmetry to continue rotating in a clockwise or counterclockwise direction toward the vertical. As the interface rotates, its elongation rate slows down until it stalls completely and then ultimately switches to contraction as it becomes more vertical (Fig. 3 D). It is interesting to note, however, that the transition or “stall” angle is not perfect at 45°, but closer to 30° (shifted toward the vertical direction) and may change over developmental time during GBE (Fig. 1 B).

**Figure 7.**
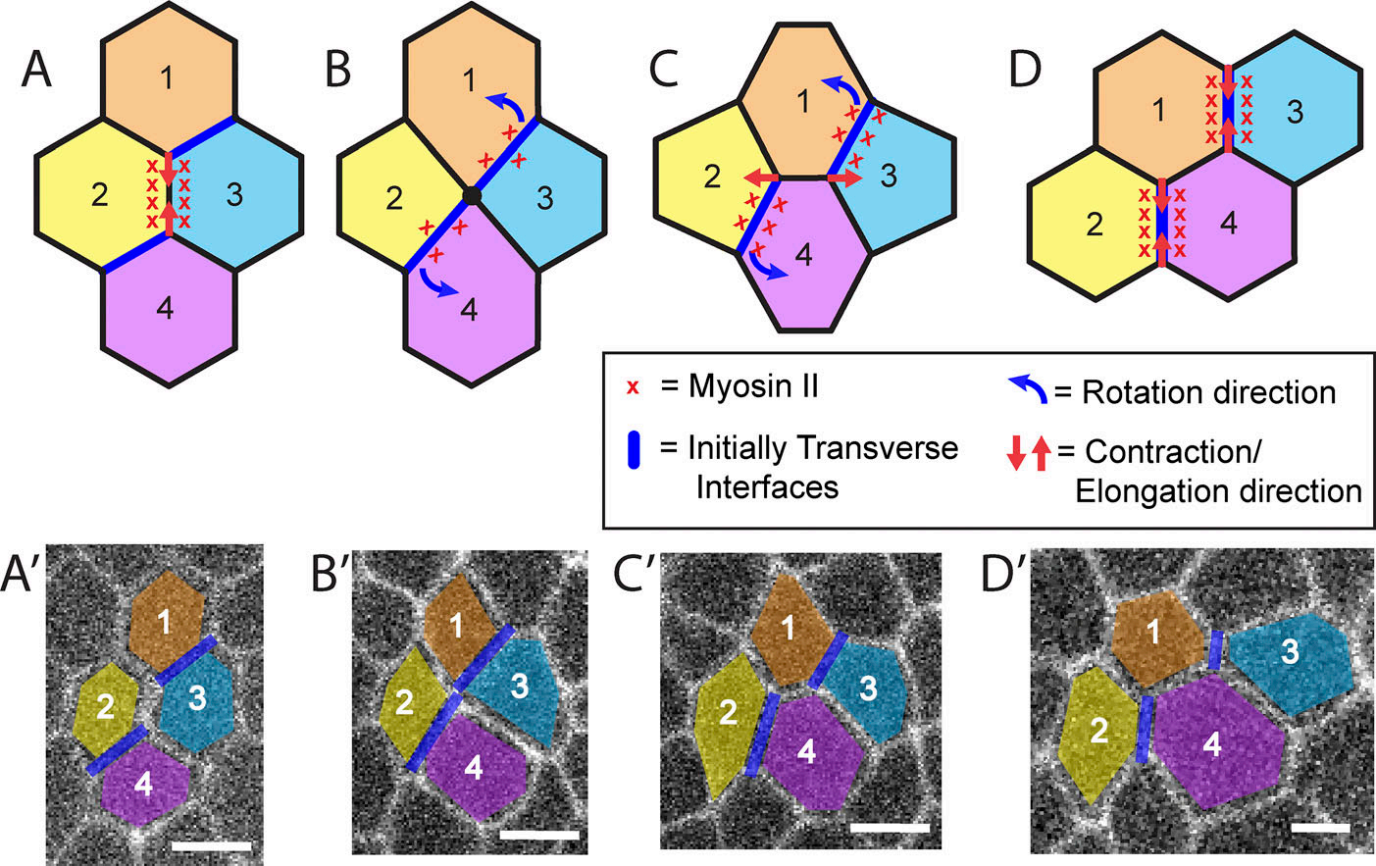
**T1 transitions contribute to systematic interface rotation.** For each model panel, we show an associated reference real-cell image from the same image data as Video 4. **(A)** Four cells (1, 2, 3, and 4) in the initial state. The vertical interface is rich in myosin and as such begins to contract. **(B)** The same four cells, now at the stage of the higher-order vertex. As the initial vertical interface contracts, it pulls along with it the transverse interfaces around it, causing them to become slightly more vertical in their orientation. As this occurs, myosin is recruited to the formerly transverse interface. **(C)** The newly formed horizontal interface elongates, which further rectifies the transverse interfaces of interest. **(D)** Cells 1, 2, 3, and 4 have now fully intercalated and have formed new cell-cell contacts (i.e., between cells 1 and 4, which originally had no contact with each other). The previously transverse interfaces of interest have now rotated to become entirely vertical, and as such represent newly formed vertical interfaces ready to undergo an additional round of intercalation.

The basic features of our model are illustrated in a small graphical schematic (Fig. 7, A and A′). A sample cell group starts out in the “canonical” hexagonal configuration, where the central vertical interface is rich in myosin (indicated by red x’s) and begins to contract for a t1 transition. However, we also explicitly track the transverse interfaces (shown in blue) over time. During the shortening of the central vertical interface, all connected transverse interfaces (blue) undergo a small rotation towards vertical orientation, which is continued as the higher-order vertex is formed (Fig. 7, B and B′) and then resolves into a horizontal interface (Fig. 7, C and C′). The rotation prompts the transverse interfaces to recruit myosin and allows them to begin contracting (Fig. 7, D and D′). As a result, the rotated transverse interfaces of interest can subsequently behave as new contracting vertical interfaces, allowing the cycling mechanism to continue.

There is no consensus in the field on whether and to what degree planar polarity and properties of individual interfaces are dynamic in time. In the “differential identity model,” planar polarity (of Myosin II and Par-3 localization) is thought to be oriented by pair-rule patterning genes so that myosin is concentrated at those interfaces between neighboring cell pairs that differ in their PRG expression identity (Zallen and Wieschaus, 2004; Bertet et al., 2004). In this model, Myosin II enrichment should be primarily dependent on the type of the interface (i.e., the PRG expression identities of the cells on either side of the interface), as opposed to its orientation (Tetley et al., 2016). This model would thus predict that the myosin status—and behavior–of a given interface should be insensitive to rotation. However, other studies have reported that the spatial anisotropy of myosin appears to be robustly dependent on the planar angle and is spatially fixed to the DV direction during the first ∼25 min of GBE (Kasza et al., 2014; Tetley et al., 2016; Farrell et al., 2017; Lefebvre et al., 2023). Our data are consistent with these latter findings since in our data, myosin localization—and the associated contraction/elongation behaviors—are not permanent properties of interfaces, but change with orientation angle, so that planar polarity of myosin and behavior persists even on those timescales over which significant topological remodeling due to intercalation occurs. While the precise angle at which an individual interface acquires myosin during its rotation is somewhat variable in time, the planar polarity as a whole is preserved over long periods of time, where the maximum myosin intensity always occurs in most vertical interfaces.

Consistent with our findings above, Farrell et al. (2017) reported that myosin II and Par-3 dynamically remodel on interfaces as cells move, increasing or decreasing their intensity as interfaces rotate. Interestingly, they also briefly noted that they observed substantially more interfaces that rotate from horizontal orientation to transverse than from vertical to transverse, although they attributed this finding to a potential sampling bias since vertical interfaces likely “contracted and disappeared before they could rotate.” We suggest that their finding was likely not a sampling artifact since it is consistent with our observation of a systematic preferential rotation direction from horizontal to vertical.

We have shown here that the planar polarized identity of an interface is not a permanent feature established once at the onset of GBE but rather a behavior that exists on a temporally dynamic continuum. Specifically, interface contractility is not limited to interfaces that are vertical at the start of germband extension. Contractility can be acquired over time by non-vertical interfaces as they rotate toward the vertical, meaning that even interfaces that are horizontal/elongating at the onset of GBE can flip their behavior and begin contracting at a later time point. Thus, contraction and rotation work together in a looping mechanism, where newly created horizontal interfaces rotate to ultimately become transverse, and transverse interfaces rotate to become vertical. If sufficient time is available, a new horizontal interface created by a t1 transition could theoretically rotate toward the vertical and fully contract to undergo another t1 transition. This cycling mechanism allows for intercalation to occur not just for the initial set of vertical interfaces—i.e., those that are vertical at the onset of germband extension—but on an ongoing basis so that more than two interfaces per cell can undergo t1 transitions. Thus, it also contributes to the phenomenon that the experimentally observed total elongation of the germband is higher than a single round of intercalation would theoretically allow (Blankenship et al., 2006). In addition, it also directly suggests a mechanism for generating multicellular rosettes. Further, our analyses show that the rotation of interfaces—and their incremental acquisition of contraction behavior—is associated with incremental myosin recruitment, and we have shown that disrupting the force-generating molecular machinery (namely Myosin and F-actin) has concurrent effects on interface rotation.

Notably, a recent live-cell study that performed full-embryo cartography of PRG expression patterns and myosin II expression (Lefebvre et al., 2023) failed to find a predictive spatial correlation between PRG and myosin II expression and reported that, over the course of germband extension, the pattern of PRG expression, in fact, started to measurably diverge from the pattern of myosin localization anisotropy in some regions of the embryo. This study proposed that myosin is recruited by a “geometrically static” source within the embryo consistent with mechanosensitive mechanisms (also see Fernandez-Gonzalez et al., 2009; Farrell et al., 2017; Gustafson et al., 2022), rather than PRG expression patterns. Importantly, our findings that myosin recruitment and contraction are dynamic behaviors (i.e., interfaces can acquire both myosin and contractile ability over time, and they specifically do so when they rotate toward vertical orientation) are hard to reconcile with any model where interface behaviors are determined by fixed cellular identities. Instead, our data are more consistent with a model where global cues (mechanical or otherwise) set the coordinate system for planar orientation. For example, it is conceivable that rotation toward the vertical exposes interfaces to higher tension or stretching, which then leads to mechanically induced myosin recruitment (Fernandez-Gonzalez et al., 2009; Gustafson et al., 2022), where the myosin recruitment then in turn initiates a bias toward contraction.

Overall, in this work, we propose a dynamic “angle rectification” mechanism that produces systematic rotation of interfaces from horizontal toward vertical orientation. During this rectification, the interfaces’ planar polarities and behaviors are constantly re-interpreted based on their current orientation angle. This cycling behavior can produce multiple “rounds” of t1 transitions in a given cell and thus contributes to increasing the macroscopic elongation of the germband.

## Materials and methods

### Fly stocks and genetics

Stocks were kept at 25°C and maintained by standard procedures. Fly stocks used in this study were 117;95-1 (Resille:GFP and Spider:GFP, cell outline markers); Gap43:mCh (a cell outline marker) and E-cad:GFP (a marker of cell junctions); and sqh:mCh (Myosin light chain marker). Torso mutants were similarly marked with Resille:GFP and Spider:GFP cell outline markers, in addition to having a loss-of-function mutation of the torso-like protein coding gene (tsl).

### Live imaging

Embryos were collected on apple juice agar and dechorionated in 50% bleach for 2 min, then rinsed with water and either staged on apple juice agar or transferred to a gas permeable microscope slide and covered with Halocarbon 27 oil. All imaging was performed at 25°C on a CSU10b Yokogawa spinning disk confocal from Zeiss and Solamere Technologies Group with a 63×/1.4 NA objective and with a Hamamatsu ORCA EMCCD camera and Micro-Manager software. The resultant time-resolved movies were positioned to capture the anterior-most portion of the germband, ∼3–5 cells posterior to the cephalic furrow and ∼3–5 cells dorsal to the ventral furrow.

### Drug injections

Following dechorionation, as previously described, embryos were staged and aligned on apple juice agar, glued to a coverslip with heptane glue, and desiccated. Embryos were covered with Halocarbon 700 oil and then injected with appropriate drug injections, as detailed in Table 1. Embryos at the beginning of germband extension were injected in the perivitelline space at 50% egg length.

**Table 1.** Drug treatment reagents and concentrations

Drug	Description	Vehicle	Final concentration
Y-27632 (Cat#281642; Santa Cruz)	RoK inhibitor (Myosin)	H_2_O	25 and 100 mM
CK666 (Cat#3950; TOCRIS)	Arp2/3 inhibitor	DMSO diluted 1:20 in H_2_O	250 µm
SMIFH2 (Cat#4401; TOCRIS)	Formin inhibitor (Diaphanous)	DMSO diluted 1:50 in H_2_O	50 µm

Note: For the most severe inhibition we used (Y-27632 100 mM), the ventral furrow fails to form. Otherwise, mesoderm invagination proceeds as normal in all the backgrounds we used.

### Repeatability

All measurements were quantified from a minimum of three embryos and represented at least two individual trials.

### Cell segmentation

Image and data analysis were performed in MATLAB (MathWorks). Cells were segmented using a seeded watershed algorithm and tracked in time. Cell areas were measured as the sum of the pixels within the contour of the watershed segmentation lines.

### Myosin intensity analysis

ROIs (regions of interest) for junctional intensity measurements were automatically generated from the tissue segmentation results for each time point. The junctional ROI was generated by computing the distance transform of the segmented interface (cell–cell junction) and constraining it to all pixels within a distance of four pixels (∼0.6 μm). Myosin intensity was defined as the ROI mean intensity, which was calculated on maximum projections of between 6 and 10 apical z-layers. To correct for photobleaching, the intensity was then normalized by subtracting the mean and dividing by the standard deviation of the entire image.

### Interface lengths, angles, and rates

Interface lengths were measured as the Euclidean distance between vertices, where vertices are the positions where three or more cells meet. Interface angles were then calculated from these vertex-bound line segments and corrected for any offset of the embryo’s body axis with respect to the image coordinate system (see below). By our definition, 0° corresponds to vertical (or DV axis aligned), ±90° corresponds to horizontal (or anterior-posterior axis aligned) in our coordinate system, and intermediate angles between these extremes are considered to be transverse. Due to the pixelated nature of the watershed transform, interface lengths are measured with a precision of single pixels and noisy variations in length are present due to confocal imaging and its interpretation by the watershed algorithm. Rates of length change were defined as: r(t)=[l(t+Δt)−l(t)]/Δt and rates of orientation angle change were defined as r(t)=[θ(t+Δt)−θ(t)]/Δt where ∆t = 30 s.

### Interface length ratchet analysis

The instantaneous cell oscillation phase is acquired from cell area oscillations using the Osculating Circle Method (Hsu et al., 2011). A more detailed explanation of the processing to acquire the cell phase can be found in (Vanderleest et al., 2018). In our implementation, the phase angles *ϕ* are related to the area oscillation time courses A(*ϕ*) as: A(ϕ)=b−c∙cos(ϕ), with constants b and c, i.e., a negative cosine function. This means conceptually that a phase angle of *ϕ* = 180° correspond to the time point of the area maximum and that *ϕ* = 0° and *ϕ* = 360° corresponds to the area minimum; therefore, the phase point corresponding to the fastest rate of contraction (area decrease) is *ϕ* = 270° and the fastest rate of expansion (area increase) at *ϕ* = 90°.

For the ratchet analysis (Fig. 1 E), which represents length change ∆L as a function of oscillation phase *ϕ*, ∆L(*ϕ*), was defined as the change relative to the start of the cycle such that ∆L = 0 for *ϕ* = 0°. Due to the fact that cell area oscillations have varying periods (and thus different numbers of time points), the length change for each cycle was interpolated to a common phase vector with 9° intervals between 0° and 360°, resulting in an ∆L vector of 40 phase points. The ∆L(*ϕ*) trajectories for all interfaces that were present for an entire cycle were averaged in a phase-resolved manner.

### Statistics

For all box-and-whisker plots (MATLAB statistics toolbox box plot), the central mark indicates the median, and bottom and top edges represent the 25th and 75th percentiles, respectively. The whiskers represent the extreme values not including outliers, where outliers are defined as being >1.5 times the interquartile range from the bottom or top of the box. Statistical significance of the differences between distributions was determined using a two-tailed *t* test unless otherwise indicated.

### Determination of start of GBE and orientation offset angle of the embryo

For each movie, the reference time point (i.e., the time point of the onset of germband extension t_0_) and the reference angle (i.e., the orientation angle corresponding to the direction of the AP and DV axes in the image) were determined through the examination of the individual heatmaps of interfaces’ length change rate vs time and orientation angle, analogous to the one shown in Fig. 1 B. In the heatmap, the first time point that produces a robust planar polarity of behavior (contraction of vertical and elongation of horizontal interfaces) is defined as the start point of germband extension; in addition, the orientation angle that maximizes contraction speed is defined as representing the local orientation angle of the DV axis direction within the image.

### Online supplemental material

Fig. S1 provides information on the angles of contracting and elongating interfaces as well as distribution of the data in Fig. 1 for certain regions in time or phase. Fig. S2 provides the individual rate and angle as functions of time for the examples in Fig. 2, A and B, and shows the evolution of a population of initially vertical interfaces over a time window of 15 min. Fig. S3 shows the boxplots of distributions for data used in Fig. 3, A–C. Fig. S4 gives data on additional alterations to the embryo, specifically a higher concentration of Y27532 injection and torso mutants. Fig. S5 gives the change in initial vertical interfaces over time, boxplot distribution from Fig. 5 D, an additional example interface with data shown as in Fig. 5, B and C, and a heatmap with interface angle, myosin intensity, and length change rates. Table S1 provides the P values for the comparison of the groups in Fig. 1 C′, using first an ANOVA analysis followed by Tukey–Kramer test, and as an alternative a Kruskal–Wallis followed by Dunn–Sidak test; and the P values for two-sided *t* tests done for the shaded areas in Fig. S1, B–C′. Table S2 shows the power law exponent for Fig. 3 A, as well as P values for two-sided *t* tests done for Fig. S3, A–C. Table S3 shows P values for a two-sided *t* test done for Fig. S5 B. Table S4 gives statistics for cells with ≤2 or ≥3 interface transition, with standard deviation, for Fig. 6 A. Video 1 shows a sample cell with color-coded interfaces and a plot of the changing orientation angle of these interfaces. Videos 2 and 3 show feasible trajectories of interfaces through time (streamlines), relating to the quiver plot in Fig. 3 D. Video 4 shows the trajectories of interfaces marked by initial orientation angle, complementary to the first frame given in Fig. 6 C.

## Supplementary Material

Table S1 provides the P values for the comparison of the groups in Fig. 1 C′, using first an ANOVA analysis followed by Tukey–Kramer test, and as an alternative a Kruskal–Wallis followed by Dunn–Sidak test; and the P values for two-sided *t* tests done for the shaded areas in Fig. S1, B–C′.

Table S2 shows the power law exponent for Fig. 3 A, as well as P values for two-sided *t* tests done for Fig. S3, A–C.

Table S3 shows P values for a two-sided *t* test done for Fig. S5 B.

Table S4 gives statistics for cells with ≤2 or ≥3 interface transition, with standard deviation, for Fig. 6 A.

## Data Availability

The primary imaging data used for this study, as well as the various MATLAB-based codes for the image and data analysis, is freely available from the corresponding authors upon reasonable request.
