# Supplementary material for: Rectification of planar orientation angle switches behavior and replenishes contractile junctions
Source: J Cell Biol. 2025 Jan 23;224(4):e202309069. doi: 10.1083/jcb.202309069 (PMC11756375; doi:10.1083/jcb.202309069)
Supplement: Table S1 — provides the P values for the comparison of the groups in Fig. 1 C′, using first an ANOVA analysis followed by Tukey–Kramer test, and as an alternative a Kruskal–Wallis followed by Dunn–Sidak test; and the P values for two-sided t tests done for the shaded areas in Fig. S1, B–C′. [file jcb_202309069_tables1.docx]

Data Tables for Figure S1:

**Figure 1C & C’:** Statistical tests for data in Figure 1C & C’

1 - ANOVA followed by Tukey-Kramer

ANOVA table

| 'Source' | 'SS' | 'df' | 'MS' | 'F' | 'Prob>F' |
| --- | --- | --- | --- | --- | --- |
| 'Groups' | 14404.4373 | 5 | 2880.88746 | 11115.0745 | 0 |
| 'Error' | 69089.5166 | 266562 | 0.25918742 | [] | [] |
| 'Total' | 83493.9539 | 266567 | [] | [] | [] |

Tukey-Kramer table

| Group A | Group B | Lower Bound | Estimate | Upper Bound | P value |
| --- | --- | --- | --- | --- | --- |
| 1 | 2 | -0.1136702 | -0.1053499 | -0.0970296 | 0 |
| 1 | 3 | -0.3202245 | -0.3108487 | -0.3014729 | 0 |
| 1 | 4 | -0.4466059 | -0.4373621 | -0.4281183 | 0 |
| 1 | 5 | -0.5479357 | -0.5382394 | -0.5285431 | 0 |
| 1 | 6 | -0.6076473 | -0.5981465 | -0.5886457 | 0 |
| 2 | 3 | -0.2161987 | -0.2054989 | -0.194799 | 0 |
| 2 | 4 | -0.3425966 | -0.3320122 | -0.3214279 | 0 |
| 2 | 5 | -0.4438713 | -0.4328896 | -0.4219078 | 0 |
| 2 | 6 | -0.5036062 | -0.4927966 | -0.4819871 | 0 |
| 3 | 4 | -0.1379461 | -0.1265134 | -0.1150806 | 0 |
| 3 | 5 | -0.2391923 | -0.2273907 | -0.2155891 | 0 |
| 3 | 6 | -0.2989393 | -0.2872978 | -0.2756563 | 0 |
| 4 | 5 | -0.1125743 | -0.1008774 | -0.0891804 | 0 |
| 4 | 6 | -0.1723199 | -0.1607844 | -0.149249 | 0 |
| 5 | 6 | -0.0718082 | -0.0599071 | -0.0480059 | 0 |

2- Krustal-Wallis followed by Dunn’s test

Krustal-Wallis table

| 'Source' | 'SS' | 'df' | 'MS' | 'Chi-sq' | 'Prob>Chi-sq' |
| --- | --- | --- | --- | --- | --- |
| 'Groups' | 3.044E+14 | 5 | 6.088E+13 | 51407.3032 | 0 |
| 'Error' | 1.27E+15 | 266562 | 4779525779 | [] | [] |
| 'Total' | 1.58E+15 | 266567 | [] | [] | [] |

Dunn-Sidak table

| Group A | Group B | Lower Bound | Estimate | Upper Bound | P value |
| --- | --- | --- | --- | --- | --- |
| 1 | 2 | -15291.99 | -13999.925 | -12707.86 | 0 |
| 1 | 3 | -44679.303 | -43223.326 | -41767.349 | 0 |
| 1 | 4 | -64185.916 | -62750.443 | -61314.971 | 0 |
| 1 | 5 | -80250.827 | -78745.082 | -77239.336 | 0 |
| 1 | 6 | -88227.909 | -86752.519 | -85277.13 | 0 |
| 2 | 3 | -30884.989 | -29223.401 | -27561.813 | 0 |
| 2 | 4 | -50394.169 | -48750.519 | -47106.868 | 0 |
| 2 | 5 | -66450.523 | -64745.157 | -63039.79 | 0 |
| 2 | 6 | -74431.219 | -72752.594 | -71073.97 | 0 |
| 3 | 4 | -21302.512 | -19527.118 | -17751.724 | 0 |
| 3 | 5 | -37354.435 | -35521.756 | -33689.077 | 0 |
| 3 | 6 | -45337.015 | -43529.194 | -41721.372 | 0 |
| 4 | 5 | -17811.07 | -15994.638 | -14178.206 | 0 |
| 4 | 6 | -25793.424 | -24002.076 | -22210.727 | 0 |
| 5 | 6 | -9855.5763 | -8007.4374 | -6159.2984 | 0 |

**Figure S1B:** p-values for a pairwise two sample t-test for distributions in Figure S1B, corresponding to the grey shaded area t = 4-6 min in Figure 1D.

| angles | 0-15 | 15-30 | 30-45 | 45-60 | 60-75 | 75-90 |
| --- | --- | --- | --- | --- | --- | --- |
| 0-15 |  | <10^-3^ | <10^-3^ | <10^-3^ | <10^-3^ | <10^-3^ |
| 15-30 |  |  | <10^-3^ | <10^-3^ | <10^-3^ | <10^-3^ |
| 30-45 |  |  |  | 7.0*10^-3^ | 0.047 | <10^-3^ |
| 45-60 |  |  |  |  | 0.28 | <10^-3^ |
| 60-75 |  |  |  |  |  | <10^-3^ |
| 75-90 |  |  |  |  |  |  |

**Figure S1B’:** p-values for a pairwise two sample t-test for distributions in Figure S1B’, corresponding to the grey shaded area t = 19-21 min in Figure 1D

| angles | 0-15 | 15-30 | 30-45 | 45-60 | 60-75 | 75-90 |
| --- | --- | --- | --- | --- | --- | --- |
| 0-15 |  | <10^-3^ | <10^-3^ | <10^-3^ | <10^-3^ | <10^-3^ |
| 15-30 |  |  | <10^-3^ | <10^-3^ | <10^-3^ | <10^-3^ |
| 30-45 |  |  |  | <10^-3^ | <10^-3^ | <10^-3^ |
| 45-60 |  |  |  |  | <10^-3^ | <10^-3^ |
| 60-75 |  |  |  |  |  | <10^-3^ |
| 75-90 |  |  |  |  |  |  |

**Figure S1C:** p-values for a pairwise two sample t-test for distribution in Figure S1C, corresponding to the grey shaded area @ ϕ=180° in Figure 1E

| angle | 0-15 | 15-30 | 30-45 | 45-60 | 60-75 | 75-90 |
| --- | --- | --- | --- | --- | --- | --- |
| 0-15 |  | <10^-3^ | <10^-3^ | <10^-3^ | <10^-3^ | <10^-3^ |
| 15-30 |  |  | <10^-3^ | <10^-3^ | <10^-3^ | <10^-3^ |
| 30-45 |  |  |  | 0.0014 | <10^-3^ | <10^-3^ |
| 45-60 |  |  |  |  | <10^-3^ | <10^-3^ |
| 60-75 |  |  |  |  |  | <10^-3^ |
| 75-90 |  |  |  |  |  |  |

**Figure S1C’:** p-values for a pairwise two sample t-test for distribution in Figure S1C’, corresponding to the grey shaded area @ ϕ=360° in Figure 1E

| angle | 0-15 | 15-30 | 30-45 | 45-60 | 60-75 | 75-90 |
| --- | --- | --- | --- | --- | --- | --- |
| 0-15 |  | <10^-3^ | <10^-3^ | <10^-3^ | <10^-3^ | <10^-3^ |
| 15-30 |  |  | <10^-3^ | <10^-3^ | <10^-3^ | <10^-3^ |
| 30-45 |  |  |  | <10^-3^ | <10^-3^ | <10^-3^ |
| 45-60 |  |  |  |  | <10^-3^ | <10^-3^ |
| 60-75 |  |  |  |  |  | 0.0022 |
| 75-90 |  |  |  |  |  |  |
