# Supplementary material for: Rectification of planar orientation angle switches behavior and replenishes contractile junctions
Source: J Cell Biol. 2025 Jan 23;224(4):e202309069. doi: 10.1083/jcb.202309069 (PMC11756375; doi:10.1083/jcb.202309069)
Supplement: Table S2 — shows the power law exponent for Fig. 3 A, as well as P values for two-sided t tests done for Fig. S3, A–C. [file jcb_202309069_tables2.docx]

Data Tables for Figure S3:

Power law exponent value (γ) for Angle MSD in Figure 3A

| Interface Angle (°) | Power Law Exponent γ |
| --- | --- |
| 0-15 | 1.094 |
| 15-30 | 1.176 |
| 30-45 | 1.289 |
| 45-60 | 1.415 |
| 60-75 | 1.205 |
| 75-90 | 1.041 |

p-values for a pairwise two sample t-test in Figure S3A (MSD)

| angles | 0-15 | 15-30 | 30-45 | 45-60 | 60-75 | 75-90 |
| --- | --- | --- | --- | --- | --- | --- |
| 0-15 |  | <10-3 | <10^-3^ | <10^-3^ | <10^-3^ | <10^-3^ |
| 15-30 |  |  | 8.5*10^-3^ | <10^-3^ | 0.071 | 0.33 |
| 30-45 |  |  |  | 0.097 | 0.37 | 1.6*10^-3^ |
| 45-60 |  |  |  |  | 0.015 | <10^-3^ |
| 60-75 |  |  |  |  |  | 6.2*10^-3^ |
| 75-90 |  |  |  |  |  |  |

p-values for a pairwise two sample t-test for Figure S3B (Length Rate)

| angle | -45 | 0 | 45 | 90 |
| --- | --- | --- | --- | --- |
| -45 |  | <10^-3^ | <10^-3^ | <10^-3^ |
| 0 |  |  | <10^-3^ | <10^-3^ |
| 45 |  |  |  | <10^-3^ |
| 90 |  |  |  |  |

p-values for a pairwise two sample t-test for Figure S3C (Rotation Rate)

| angle | -45 | 0 | 45 | 90 |
| --- | --- | --- | --- | --- |
| -45 |  | <10^-3^ | <10^-3^ | <10^-3^ |
| 0 |  |  | <10^-3^ | <10^-3^ |
| 45 |  |  |  | <10^-3^ |
| 90 |  |  |  |  |
