# Supplementary material for: Rectification of planar orientation angle switches behavior and replenishes contractile junctions
Source: J Cell Biol. 2025 Jan 23;224(4):e202309069. doi: 10.1083/jcb.202309069 (PMC11756375; doi:10.1083/jcb.202309069)
Supplement: Table S3 — shows P values for a two-sided t test done for Fig. S5 B. [file jcb_202309069_tables3.docx]

Data Tables for Figure S5:

p-values for pairwise two sample t-test for Figure S5B (Myosin Intensity)

| Timepoint | first 30 seconds | middle 30 seconds |  | last 30 seconds |
| --- | --- | --- | --- | --- |
| first 30 seconds |  |  | <10^-3^ | <10^-3^ |
| middle 30 seconds |  |  |  | <10^-3^ |
| last 30 seconds |  |  |  |  |
