# Supplementary material for: Rectification of planar orientation angle switches behavior and replenishes contractile junctions
Source: J Cell Biol. 2025 Jan 23;224(4):e202309069. doi: 10.1083/jcb.202309069 (PMC11756375; doi:10.1083/jcb.202309069)
Supplement: Table S4 — gives statistics for cells with ≤2 or ≥3 interface transition, with standard deviation, for Fig. 6 A. [file jcb_202309069_tables4.docx]

Statistics for percentage of cells with ≤2 or ≥3 interface transition, with standard deviation, for Figure 6A

| Time Interval  How Long Cells are  Visible (minutes) | Percentage of Cells with ≤2 Interface  Transitions | Percentage of Cells with ≥3 Interface  Transitions | Standard  Deviation (n=3) |
| --- | --- | --- | --- |
| 0-5 | 99.56 | 0.44 | 0.35 |
| 5-10 | 91.67 | 8.33 | 5.32 |
| 10-15 | 79.58 | 20.42 | 14.39 |
| 15-20 | 67.22 | 32.78 | 17.13 |
| 20-25 | 62.85 | 37.15 | 30.60 |
